# Healthy aging meta-analyses and scoping review of risk factors across Latin America reveal large heterogeneity and weak predictive models

**DOI:** 10.1038/s43587-024-00648-6

**Published:** 2024-06-17

**Authors:** Agustin Ibanez, Marcelo Maito, Felipe Botero-Rodríguez, Sol Fittipaldi, Carlos Coronel, Joaquin Migeot, Andrea Lacroix, Brian Lawlor, Claudia Duran-Aniotz, Sandra Baez, Hernando Santamaria-Garcia

**Affiliations:** 1 Latin American Brain Health Institute (BrainLat), Universidad Adolfo Ibañez, Santiago de Chile, Chile; 2 Global Brain Health Institute (GBHI), University of California, San Francisco (UCSF), San Francisco, CA USA; 3 University of Trinity Dublin, Dublin, Ireland; 4 Cognitive Neuroscience Center (CNC), Universidad de San Andrés, Buenos Aires, Argentina; 5 Trinity College Dublin, Dublin, Ireland; 6 PhD Program of Neuroscience, Department of Psychiatry, Pontificia Universidad Javeriana, Bogotá, Colombia; 7 Hospital Universitario San Ignacio, Center for Brain and Cognition, Intellectus, Bogotá, Colombia; 8 Fundación para la Ciencia, Innovación y Tecnología – Fucintec, Bogotá, Colombia; 9 Centro Interdisciplinario de Neurociencia de Valparaíso (CINV), Universidad de Valparaíso, Valparaíso, Chile; 10 Herbert Wertheim School of Public Health and Human Longevity Science, Health Sciences Office of Faculty Affairs, University California, San Diego (UCSD), San Diego, CA USA; 11 Universidad de los Andes, Bogotá, Colombia

**Keywords:** Medical research, Databases, Ageing

## Abstract

Models of healthy aging are typically based on the United States and Europe and may not apply to diverse and heterogeneous populations. In this study, our objectives were to conduct a meta-analysis to assess risk factors of cognition and functional ability across aging populations in Latin America and a scoping review focusing on methodological procedures. Our study design included randomized controlled trials and cohort, case–control and cross-sectional studies using multiple databases, including MEDLINE, the Virtual Health Library and Web of Science. From an initial pool of 455 studies, our meta-analysis included 38 final studies (28 assessing cognition and 10 assessing functional ability, *n* = 146,000 participants). Our results revealed significant but heterogeneous effects for cognition (odds ratio (OR) = 1.20, *P* = 0.03, confidence interval (CI) = (1.0127, 1.42); heterogeneity: *I*^2^ = 92.1%, CI = (89.8%, 94%)) and functional ability (OR = 1.20, *P* = 0.01, CI = (1.04, 1.39); *I*^2^ = 93.1%, CI = (89.3%, 95.5%)). Specific risk factors had limited effects, especially on functional ability, with moderate impacts for demographics and mental health and marginal effects for health status and social determinants of health. Methodological issues, such as outliers, inter-country differences and publication bias, influenced the results. Overall, we highlight the specific profile of risk factors associated with healthy aging in Latin America. The heterogeneity in results and methodological approaches in studying healthy aging call for greater harmonization and further regional research to understand healthy aging in Latin America.

## Abstract (web summary)

Ibanez et al. performed a scoping review and meta-analysis of healthy aging studies across Latin America and report substantial heterogeneity in how risk factors affect cognitive and functional ability, underscoring the need for further regional research.

## Main

The understanding of healthy aging and brain health has been informed primarily by studies from the United States and Europe^1–5^. These studies predominantly focus on cognitive abilities (that is, attention, problem-solving, learning and memory, among others) and functional abilities (that is, specific personal activities of daily living and higher-order instrumental skills), respectively^6–8^. Models are considered generalizable, as relatively small divergences in the aging process, irrespective of geographic or socioeconomic factors, have been assumed^2,9,10^. Previous reviews and meta-analyses in the United States and Europe reported consistent predictors of cognition and functional ability, including demographics (age and gender), cardiometabolic diseases, lifestyle (sleep problems, alcohol consumption and physical activity), mental health and social determinants of health (SDH) (education and socioeconomic status), particularly for high-income countries^11–16^.

However, there is a dearth of studies from other parts of the world, especially from regions with large social and health disparities^4,9,10^. The long-standing focus on high-income countries has inadvertently created a research evidence base that lacks representation from a broader spectrum of diverse populations and regions. Recently, prediction models of aging and brain–phenotype associations developed in high-income countries have shown poor reproducibility in more diverse populations^1,2,10,17^. These results indicate non-universal patterns markedly influenced by socioeconomic disparities and demographic variables, such as age and sex^2,10,11,18^. The differences across regions emphasize the need to move away from a one-size-fits-all approach to a more region-specific, tailored understanding of healthy aging and brain health.

In Latin American countries (LACs), the current prevalence of dementia stands at 8.5%, and it is expected to increase to 19.33% by 2050 (refs. ^19,20^). Such a projection significantly exceeds the prevalence estimates for Europe and the United States^3^, highlighting the need to contextualize the risk factors of the LACs^21^, a region of large inequalities and unique racial–ethnic admixtures^9,10,20,19^. Recent studies suggest that demographic factors impact healthy aging in a varied manner in LACs. This includes (1) a more significant impact from social and health disparities compared to age and sex^1,2,5^ and (2) less pronounced effects of the latter two compared to other regions^1,5,22^. We recently reported that cognition and functional abilities in healthy aging across LACs are influenced by heterogeneous factors that are different from other regions, mainly related to social and health disparities^1^. That study suggests that the disparities reflect non-universal effects of aging, deeply influenced by the numerous disparity-related cumulative exposures^2,18^ that escalate the risks associated with aging and dementia. However, except for the above-mentioned study, the cumulative evidence has many gaps and methodological flaws^1,2,10,18,19^. There is substantial heterogeneity regarding sample sizes, designs, populations, statistical approaches and outcomes. Most studies have not evaluated the interactions between different potential risk factors^10^. The focus on individual or a small number of countries within the region, combined with the lack of harmonization, can lead to a priori biases and non-representative regional findings^20^.

**Fig. 1:**
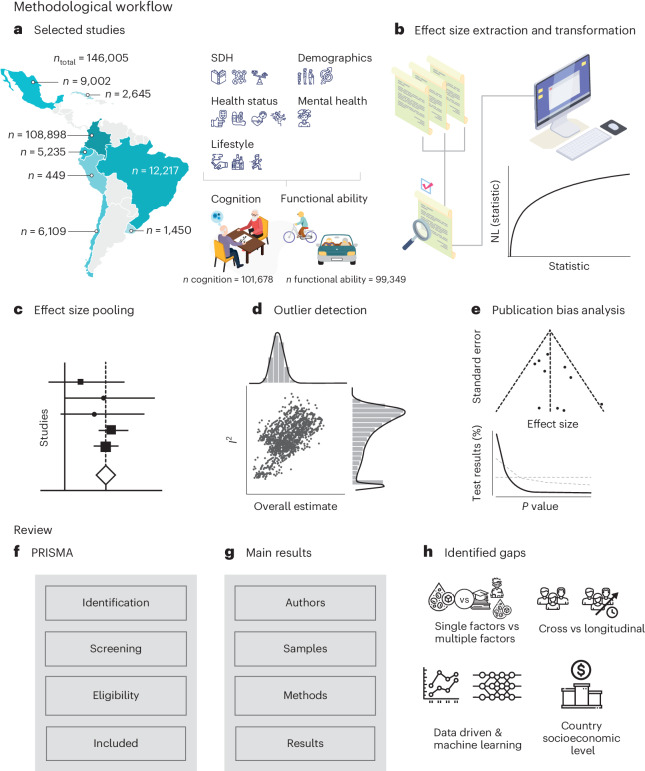
Methodological workflow. **a**–**e**, Meta-analysis. The left part of **a** (selected studies) presents the sample size (*n*) by each country. On the right side of **a**, we describe of the group of risk factors (demographic, SDH, health status, mental health and lifestyle) associated with the healthy aging outcomes (cognition and functional ability). **b** (effects size extraction and transformation) illustrates the procedure followed by three independent researchers to extract effect sizes of each risk factor for cognition and functional ability across studies. NL, natural logarithm. **c**, Effect size pooling: the panel displays the graphic representation of a forest plot, used to present pooled effect sizes of the studies assessed in this work (no real data are depicted in this panel). **d** (outlier detection) reveals the graphic representation of heterogeneity *I*^2^ across studies (*y* axis) versus the predicted overall estimate (no real data are depicted in this panel). **e**, Publication bias analysis: graphic representation of the funnel plot in the upper section and, in the lower section, a graph that depicts the strength of *P* value patterns across studies (no real data are depicted in this panel). The horizontal and diagonal dashed lines denote ‘Null of no effect’ and ‘Null of 33% power’, respectively. **f**–**h**, Scoping review. **f**, PRISMA: study stages following the PRISMA flowchart (no real data are depicted in this panel). **g** (main results) indicates the specific information extracted from the studies (no real data are depicted in this panel). **h**, Identified gaps. This panel provides the gaps identified in each study, including the inclusion of one versus many risk factors, the type of studies (cross-sectional, longitudinal or combined), the analytical approaches using data-driven insights and machine learning techniques and the consideration of countries with different socioeconomic backgrounds.

To address these gaps, we studied different factors influencing healthy aging, as reflected by cognition and functional ability across LACs, using meta-analytical and systematic review approaches (Fig. 1). We first assessed the overall effects of combined risk factors. We then performed separate meta-analyses of predictors, including demographics, health status, mental health symptoms, lifestyle factors and SDH. From an initial number of 455 studies, our meta-analyses included *n* = 146,005 participants and 38 final reports (28 for cognition and 10 for functional ability). We addressed the robustness of the results in terms of main effects, heterogeneity and multiple influences, considering different predictors, country differences, methods employed and publication bias. In addition, we performed a Preferred Reporting Items for Systematic Reviews and Meta-Analyses (PRISMA) scoping review to address additional aspects related to the methodological procedures employed.

## Results

### Meta-analysis

**Fig. 2:**
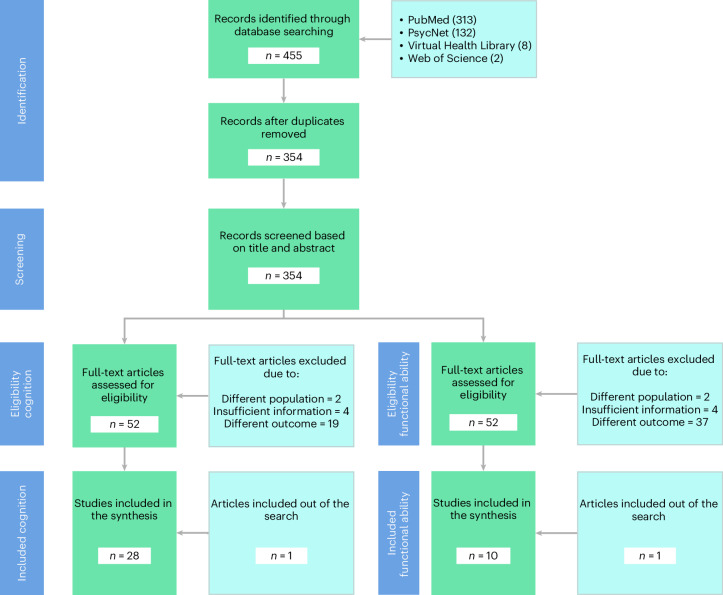
PRISMA flowchart. PRISMA methodology for searching and selecting studies. Using the search criteria, we identified 1,382 studies (PubMed 313, PsycNet 132, Virtual Health Library 8, Web of Science 2). After implementing a procedure to detect duplicates, we retained 354 studies for further analysis. Three independent evaluators assessed the relevance of the title and abstract of each study, ultimately selecting 52 studies. Four full-text articles were excluded due to insufficient or unsuitable information for our research interests, two because of a different population study and 19 for assessing different outcomes. In the end, 38 studies were selected for further analysis: 28 on risk factors for cognition and 10 on risk factors for functional ability.

A comprehensive exploration of literature published until 16 August 2023 was performed in multiple databases (including MEDLINE, Virtual Health Library and Web of Science; Fig. 2 and Supplementary File 1), leveraging a variety of keywords encompassing aging, brain health, lifestyle, demographics, mental health, SDH and other factors, specifically focusing on LACs. A team of three reviewers extracted detailed information from all the sourced articles, and the results were verified by an additional independent reviewer. Year of publication, effect size, type of effect size and control variables were obtained (data analysis section). Our research focused on five primary factors identified as critical determinants of healthy aging in previous studies^1^. These include insights from the Lancet Consortium for Dementia Prevention and Care^3^, encompassing demographics (age and sex), SDH (education, social isolation, socioeconomic status, ethnicity and race), health status (hypertension, diabetes and obesity), mental health status (depression, anxiety and stress-related disorders) and lifestyle factors (smoking, alcohol consumption and physical activity). Other factors relevant to aging were not included, given the lack of a minimum available number of reports. A complete list of studies included in all analyses for cognition^1^^,23–49^ and functional ability^1,28,50–57^ is included here and further described in Supplementary Table 1.

Egger’s test^58^ and enhanced funnel plots^59^ were used to gauge publication bias, complemented by a p-curve test assessing the empirical evidence strength and potential biases^60^. The *I*^2^ index^61^ and Graphic Display of Heterogeneity (GOSH^62^) plots provided an assessment of heterogeneity, supported by subgroup analyses across different countries (Methods). Given the existence of high heterogeneity, outlier data points were identified and excluded in further analyses to maintain data integrity^63^. Leveraging random effects models, factors were analyzed with the Paule–Mandel estimator^64^ using Knapp–Hatung adjustments^65^ to minimize false discovery rates. The age ranges in most studies were above 59 years. However, some studies included individuals aged 35 years and older. Table 1 provides a detailed description of the age ranges used across these studies, along with details of studies assessing cognition and functional ability in each country and the meta-analysis. Table 2 presents the major results of the meta-analyses of cognition, and Table 3 presents the results of the meta-analyses of functional ability.

**Table 1:** Details of studies assessing cognition and functional ability in each country and meta-analysis

		Cognition		Functional ability		Age ranges
	Country	*n* = Full	*n* = W/o outliers	*n* = Full	*n* = W/o outliers	Full
All predictors	Brazil	10,080	8,492	2,215	2,215	35–>90
	Chile	4,339	4,339	470	470	60–89
	(Colombia, Ecuador, Chile, Uruguay)	31,680	31,680	31,680	31,680	>64
	Colombia	43,933	416	64,614	41,271	60–96
	Cuba	2,645	2,645	–	–	60–>75
	Mexico	9,387	9,118	–	–	50–>65
	Peru	–	–	449	449	>59
Demographics	Brazil	4,486	3,563	1,907	1,626	35–>80
	Chile	2,955	2,955	470	470	60–89
	(Colombia, Ecuador, Chile, Uruguay)	31,680	31,680	31,680	–	>64
	Colombia	23,759	416	64,614	64,614	60–96
	Cuba	2,645	2,645	–	–	60–>75
	Mexico	1,109	1,109	–	–	>49
	Peru	–	–	449	449	>59
Health	Brazil	6,081	6,081	1,779	1,413	35–>80
	Chile	1,571	1,571	470	470	60
	(Colombia, Ecuador, Chile, Uruguay)	31,680	31,680	31,680	31,680	>64
	Colombia	23,343	–	64,614	64,614	>64
	Cuba	2,645	1,846	–	–	65–>75
	Mexico	2,416	1,826	–	–	>64
	Peru	–	–	449	449	>59
Mental health	Brazil	1,875	1,875	–	–	>59
	Chile	1,571	1,571	–	–	>59
	(Colombia, Ecuador, Chile, Uruguay)	31,680	–	31,680	31,680	>64
	Colombia	416	416	47,037	47,037	60–>80
	Cuba	1,846	1,846	–	–	65–>75
	Mexico	1,291	1,291	–	–	>54
	Peru	–	–	–	–	>59
Lifestyle	Brazil	4,964	3,376	1,413	1,413	35–>80
	Chile	2,955	1,384	–	–	>59
	(Colombia, Ecuador, Chile, Uruguay)	31,680	31,680	31,680	31,680	>64
	Colombia	43,517	23,343	47,037	47,037	>64
	Cuba	1,846	1,846	–	–	65–>75
	Mexico	4,495	4,495	–	–	>50
	Peru	–	–	–	–	>59
SDH	Brazil	5,368	3,780	2,050	2,050	35–>90
	Chile	1,384	1,384	–	–	>59
	(Colombia, Ecuador, Chile, Uruguay)	31,680	31,680	31,680	31,680	>64
	Colombia	23,759	416	64,614	41,271	60–96
	Cuba	2,645	2,645	–	–	65–>75
	Mexico	3,414	3,414	–	–	>50
	Peru	–	–	449	449	>59

**Table 2:** Meta-analyses of cognition

		*k*	OR	95% CI	*P*	*t*	95% PI	*I*^2^	95% CI
All factors	Random effects model	28	1.2006	(1.0127, 1.4234)	0.0363	2.2	(0.5912, 2.4382)	92.1%	(89.8%, 94.0%)
	Outliers removed	24	1.2651	(1.1443, 1.3987)	<0.0001	4.85	(1.0272, 1.5582)	16.9%	(0.0%, 49.4%)
Demographics	Random effects model	15	1.5098	(1.1905, 1.9147)	0.0023	3.72	(0.7144, 3.1909)	89.2%	(83.9%, 92.8%)
	Outliers removed	13	1.7386	(1.3997, 2.1595)	<0.0001	5.56	(1.0247, 2.9496)	48.2%	(1.7%, 72.7%)
Health	Random effects model	16	1.2856	(1.0136, 1.6305)	0.0397	2.25	(0.5822, 2.8385)	80.1%	(68.4%, 87.4%)
	Outliers removed	13	1.2259	(1.0601, 1.4175)	<0.0001	3.05	(0.8819, 1.7040)	70.4%	(47.9%, 83.2%)
Mental health	Random effects model	10	1.6803	(1.1848, 2.3830)	0.0084	3.36	(0.6409, 4.4051)	84.9%	(73.9%, 91.2%)
	Outliers removed	9	1.8175	(1.2699, 2.6012)	0.0049	3.84	(0.7416, 4.4543)	61.2%	(19.6%, 81.3%)
Lifestyle	Random effects model	13	1.04	(0.7765, 1.3930)	0.7747	0.29	(0.3836, 2.8197)	93.4%	(90.4%, 95.4%)
	Outliers removed	10	0.9623	(0.7490, 1.2363)	0.7364	−0.35	(0.4841, 1.9129)	83.4%	(70.9%, 90.5%)
SDH	Random effects model	15	0.9994	(0.6110, 1.6348)	0.998	0	(0.1656, 6.0331)	98.2%	(97.8%, 98.6%)
	Outliers removed	13	1.0658	(0.7997, 1.4205)	0.6373	0.48	(0.4734, 2.3996)	77.2%	(61.2%, 86.6%)

PI, prediction interval.

**Table 3:** Meta-analyses of functional ability

		*k*	OR	95% CI	*P*	*t*	95% PI	*I*^2^	95% CI
All predictors	Random effects model	10	1.2088	(1.0470,1.3956)	0.0153	2.99	(0.8248, 1.7717)	93.1%	(89.3%, 95.5%)
	Outliers removed	9	1.2795	(1.242, 1.4562)	0.0023	4.39	(0.9472, 1.7283)	75.9%	(53.8%, 87.5%)
Demographics	Random effects model	9	1.1232	(0.7593, 1.6613)	0.5132	0.68	(0.3381, 3.7311)	95.6%	(93.4%, 97.1%)
	Outliers removed	7	1.137	(0.8681, 1.4891)	0.2887	1.16	(0.5685, 2.2740)	94.8%	(91.6%, 96.8%)
Health	Random effects model	8	1.4634	(0.8708, 2.4595)	0.1264	1.73	(0.3152, 6.7956)	79.6%	(60.2%, 89.5%)
	Outliers removed	7	1.1921	(1.1260, 1.2622)	0.0003	7.53	(1.0688, 1.3298)	58.5%	(4.3%, 82%)
Mental health	Random effects model	2	1.083	(0.9878, 1.1874)	0.0577	11.01	–	–	–
	–	–	–	–	–	–	–	–	–
Lifestyle	Random effects model	4	1.3086	(0.6875, 2.4906)	0.2756	1.33	(0.2154, 7.9499)	99.4%	(99.2%, 99.6%)
	**–**	**–**	**–**	**–**	**–**	**–**	**–**	**–**	**–**
SDH	Random effects model	8	1.2273	(0.9382, 1.6055)	0.1144	1.8	(0.6113, 2.4641)	99.0%	(98.7%, 99.2%)
	Outliers removed	7	1.3401	(1.0338, 1.7371)	0.0328	2.76	(0.7314, 2.4555)	99.1%	(98.8%, 99.3%)

PI, prediction interval.

### Cognition

Our research identified 38 articles, and 28 were included in the analysis of cognition. Sample size of each meta-analysis is provided in Table 1. Supplementary Table 1 summarizes the articles and the main results.

#### All factors

**Fig. 3:**
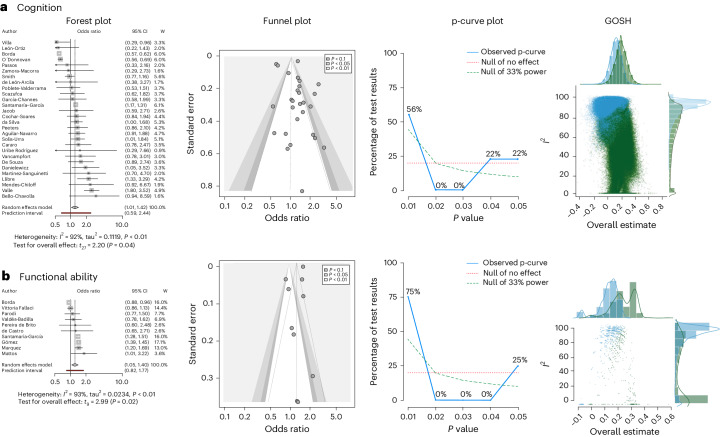
Meta-analysis across all risk factors for cognition and functional ability. **a**, Cognition. Forest plot shows *k* studies in random effects model (first author, OR, CI and weights). The random effects model results (cognition: *k* = 28, *n* = 102,064, OR = 1.2006, *P* = 0.0363, CI = (1.0127, 1.4234); functional ability: *k* = 10, *n* = 99,428, OR = 1.2088, *P* = 0.0153, CI = (1.0470, 1.3956)) are reported with Knapp–Hartung correction for false discovery rate, the prediction interval and heterogeneity values (*I*^2^ and tau^2^). W, weights. **b**, Functional ability. Forest plot shows *k* studies in random effects model (first author, OR, CI and weights). The random effects model results (functional ability: *k* = 10, *n* = 99,428, OR = 1.2088, *P* = 0.0153, CI = (1.0470, 1.3956)) are reported with Knapp–Hartung correction for false discovery rate, the prediction interval and heterogeneity values (*I*^2^ and tau^2^). For **a** and **b**, contour-enhanced funnel plot shows effect sizes, standard errors and significance; p-curve analysis shows the accumulation of *P* values over the significant studies (observed p-curve), the no-effect curve and 33% power curve; and the GOSH shows distribution for all 2^*k*−1^ possible study combinations (1 million randomly selected models when 2^*k*−1^ > 10^6^) in blue and leaving out the most negatively influential study in green.

The meta-analysis combining all factors (*k* = 28) reached statistical significance (odds ratio (OR) = 1.2006, *P* = 0.0363, confidence interval (CI) = (1.0127, 1.4234)), although pronounced heterogeneity was detected (*I*^2^ = 92.1%, CI = (89.8%, 94%)). After excluding outliers, the heterogeneity markedly decreased (*I*^2^ = 16.9%, CI = (0.0%, 49.4%); Supplementary Figs. 1–3). Concurrently, the pooled effect size exhibited enhanced statistical significance with more precise CIs (OR = 1.2651, *P* < 0.0001, CI = (1.1443, 1.3987); Table 1 and Fig. 3a). Subgroup analysis showed significant differences between countries (*P* < 0.0001), and effect sizes ranged from 0.6065 to 1.7182 (Extended Data Table 1). Egger’s test (*P* = 0.3388; Extended Data Table 2) did not indicate asymmetry (Fig. 3a). p-curve analysis showed significant results for both half and full p-curve tests, indicating the presence of a true non-zero effect (z full = −8.397, *P* < 0.001, z half = −12.92, *P* < 0.001, power estimate = 99%, CI = (98.3%, 99%); Fig. 3a and Extended Data Table 3).

#### Demographic factors

**Extended Data Fig. 1:**
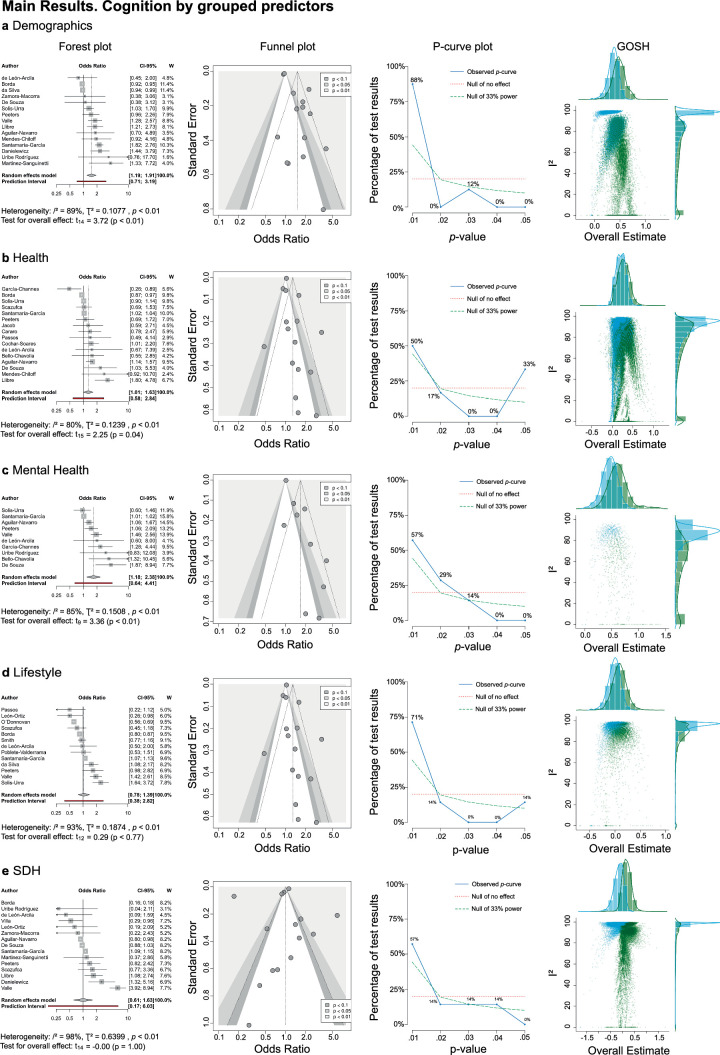
Metanalysis across different risk factors for Cognition. Each panel illustrates the major effects for each factor analyzed: panel a focuses on demographics; panel b on health; panel c on mental health; panel d on lifestyle; and panel e on Social Determinants of Health (SDH). The forest plot shows -k studies using the random effects model (first author, odds ratio, confidence interval, and weights). The random effects model results (Demographics: k = 15, n = 66634, OR = 1.5098, p-value = 0.0023, CI = [1.1905; 1.9147]; Health: k = 16, n = 67736, OR = 1.2856, p-value = 0.0397, CI = [1.0136; 1.6305]; Mental health symptoms: k = 16, n = 38679, OR = 1.6803, p-value = 0.0084, CI = [1.1848; 2.3830]; Lifestyle: k = 13, n = 89457, OR = 1.04, p-value = 0.7747, CI = [0.7765; 1.3930]; SDH: k = 15, n = 68250, OR = 0.9994, p-value = 0.998, CI = [0.6110; 1.6348]) are reported with Knapp-Hartung correction for false discovery rate, the prediction interval, and heterogeneity values (I², tau²). Contour-enhanced funnel plot showing effect sizes, standard errors, and significance. P-curve analysis, showing the accumulation of p-values over the significant studies (observed p-curve), the no-effect curve and 33% power curve. GOSH (Graphic Display of Heterogeneity) shows the distribution for all 2k-1 possible study combinations (1 million randomly selected models when 2k-1 > 106) in blue and leaving out the most negatively influential study in green.

Demographics factors (age and sex, *k* = 15) reached statistical significance (OR = 1.5098, *P* = 0.0023, CI = (1.1905, 1.9147)), with high heterogeneity (*I*^2^ = 89.2%, CI = (83.9%, 92.8%)). The pooled effect size without outliers reached statistical significance (OR = 1.2651, *P* < 0.0001, CI = (1.1443, 1.3987)), with moderate heterogeneity (*I*^2^ = 48.2%, CI = (1.7%, 72.7%); Table 1 and Extended Data Fig. 1a). Subgroup analysis showed significant differences between countries (*P* < 0.0001), and effect sizes ranged from 1.0946 to 2.2418 (Extended Data Table 1). Egger’s test (*P* = 0.0288) indicated asymmetry (Extended Data Table 2 and Extended Data Fig. 1b), and the p-curve analysis showed significant results for both half and full p-curve tests, suggesting the presence of a true non-zero effect (z full = −6.748, *P* < 0.001, z half = −6.554, *P* < 0.001, power estimate = 94%, CI = (81.5%, 98.4%); Extended Data Table 3 and Extended Data Fig. 1a).

#### Health status

The health factors meta-analysis (cardiometabolic risks and other diseases, *k* = 16) was significant (OR = 1.2856, *P* = 0.0397, 95% CI = (1.0136, 1.6305)) and revealed high heterogeneity (*I*^2^ = 80.1%, CI = (68.4%, 87.4%)). After excluding outliers, heterogeneity slightly decreased (*I*^2^ = 70.4%, CI = (47.94%, 83.2%); Supplementary Figs. 1–3). Concurrently, the pooled effect size exhibited enhanced statistical significance with narrower CIs (OR = 1.2259, *P* < 0.0001, 95% CI = (1.0601, 1.4175); Table 1 and Extended Data Fig. 1b). Subgroup analysis showed significant differences between countries (*P* = 0.0288), and effect sizes ranged from 0.9227 to 1.9227 (Extended Data Table 1). Egger’s test (*P* = 0.0926) did not indicate asymmetry (Extended Data Table 2 and Extended Data Fig. 1c). p-curve analysis showed significant results for both half and full p-curve tests, evidencing the presence of a true non-zero effect (z full = −5.848, *P* < 0.001, z half = −7.611, *P* < 0.001, power estimate = 96%, CI = (83.1%, 99%); Extended Data Table 3 and Extended Data Fig. 1b).

#### Mental health symptoms

Meta-analysis of mental health symptoms (*k* = 16) had significant effects (OR = 1.6803, *P* = 0.0084, CI = (1.1848, 2.3830)) and exhibited high heterogeneity (*I*^2^ = 84.9%, CI = (73.9%, 91.2%)). The pooled effect size without outliers was significant (OR = 1.8175, *P* = 0.0049, CI = (1.2699, 2.6012); Table 1, Extended Data Fig. 1c and Supplementary Figs. 1–3) and showed decreased heterogeneity (*I*^2^ = 61.2%, CI = (19.6%, 81.3%)). Subgroup analysis yielded significant differences between countries (*P* = 0.0006), and effect sizes ranged from 0.952 to 1.3.171 (Extended Data Table 1). Egger’s test (*P* = 0.0013) indicated asymmetry (Extended Data Table 2 and Extended Data Fig. 1d). p-curve analysis showed significant results for both half and full p-curve tests, indicating the presence of a true non-zero effect (z full = −6, *P* < 0.001, z half = −4.603, *P* < 0.001, power estimate = 94%, CI = (77.8%, 98.8%); Extended Data Table 3 and Extended Data Fig. 1c).

#### Lifestyle

The lifestyle factors metanalysis (physical activity, nutrition, alcohol consumption and smoking, *k* = 13) was not significant (OR = 1.04, *P* = 0.7747, 95% CI = (0.7765, 1.3930)) and showed high heterogeneity (*I*^2^ = 93.4%, CI = (90.4%, 95.4%)). After excluding outliers, the random effects model remained non-significant (*P* > 0.05), and heterogeneity slightly decreased (Extended Data Table 3, Extended Data Fig. 1d and Supplementary Figs. 1–3). Subgroup analysis showed significant differences between countries (*P* = 0.0173), and effect sizes ranged from 0.7225 to 1.6621 (Extended Data Table 1). Egger’s test (*P* = 0.5799) did not indicate asymmetry (Extended Data Table 2 and Extended Data Fig. 1c). p-curve analysis showed significant results for both half and full p-curve tests, indicating the presence of a true non-zero effect (z full = −9.129, *P* < 0.001, z half = −9.568, *P* < 0.001, power estimate = 99%, CI = (97.9%, 99%); Extended Data Table 3 and Extended Data Fig. 1d).

#### SDH

The SDH factors meta-analysis (educational attainment and socioeconomic status, *k* = 15) did not reach significance (OR = 0.9994, *P* = 0.998, 95% CI = (0.6110, 1.6348)) and showed high heterogeneity (*I*^2^ = 98.2%, CI = (97.8%, 98.6%)). After excluding outliers, the random effects model remained not statistically significant (*P* > 0.05), and heterogeneity (*I*^2^ = 77.2%, CI = (61.2%, 86.6%)) slightly decreased (Extended Data Table 3, Extended Data Fig. 1e and Supplementary Figs. 1–3). Subgroup analysis by country showed significant differences (*P* < 0.0001), and effect sizes ranged from 0.7225 to 1.6621 (Extended Data Table 1). Egger’s test (*P* = 0.5855) did not indicate asymmetry (Extended Data Table 2 and Extended Data Fig. 1c). p-curve analysis showed significant results for both half and full p-curve tests, indicating the presence of a true non-zero effect (z full = −8.848, *P* < 0.001, z half = −10.10, *P* < 0.001, power estimate = 99%, CI = (99%, 99%); Extended Data Table 3 and Extended Data Fig. 1e).

### Functional ability

Ten studies were included as they assessed specifically risk factors of functional ability (Supplementary Table 1 summarizes the articles and the main results). Sample size included in each meta-analysis is provided in Table 1.

#### All factors

The meta-analysis including all factors (*k* = 10) achieved significant effects (OR = 1.2088, *P* = 0.0153, CI = (1.0470, 1.3956)). However, there was pronounced heterogeneity (*I*^2^ = 93.1%, CI = (89.3%, 95.5%)). After excluding outliers, the heterogeneity remained high (*I*^2^ = 75.9%, CI = (53.8%, 87.5%); Supplementary Figs. 1–3). Concurrently, the pooled effect size exhibited enhanced statistical significance with more precise CIs (OR = 1.2795, *P* = 0.0023, CI = (1.242, 1.4562); Table 3 and Fig. 3b). Subgroup analysis by country did not show significant differences (*P* = 0.2399), and effect sizes ranged from 1.07 to 1.3861 (Extended Data Table 4). Egger’s test (*P* = 0.1884) did not indicate asymmetry (Fig. 3b and Extended Data Table 5). p-curve analysis showed significant results for both half and full p-curve tests, indicating the presence of a true non-zero effect (z full = −8.425, *P* < 0.001, z half = −10.228, *P* < 0.001, power estimate = 99%, CI = (99%, 99%); Fig. 3b and Extended Data Table 6).

#### Demographic factors

**Extended Data Fig. 2:**
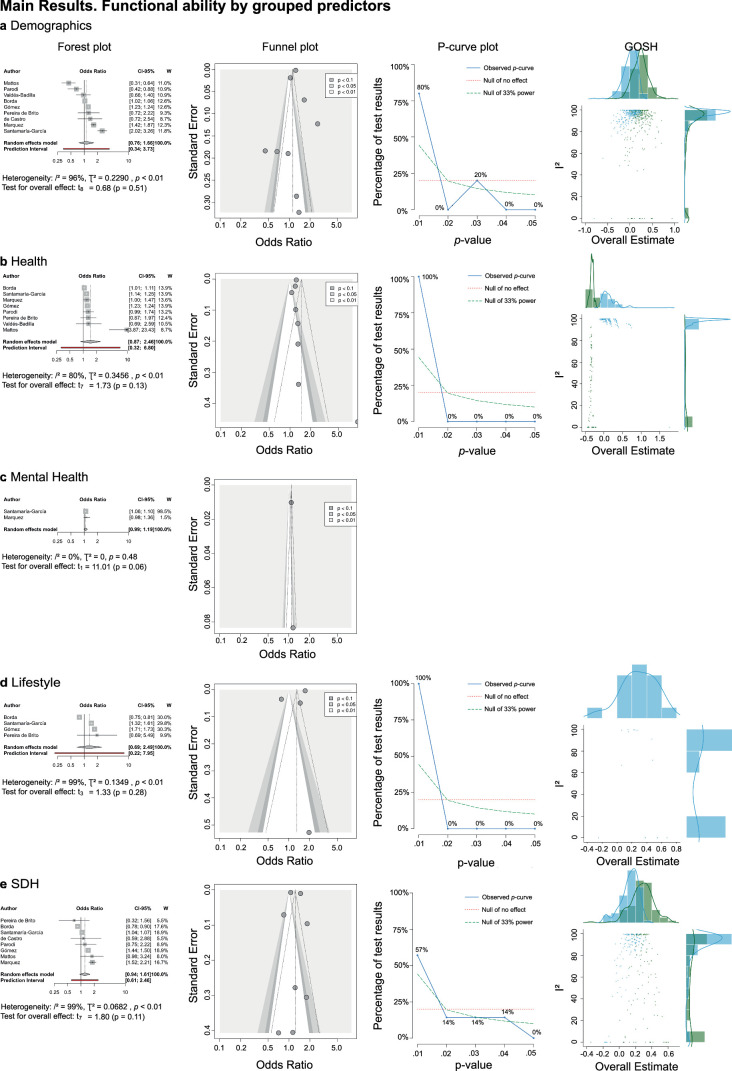
Metanalysis across different risk factors for functional ability. Each panel illustrates the major effects for each factor analyzed: panel a focuses on demographics; panel b on health; panel c on mental health; panel d on lifestyle; and panel e on Social Determinants of Health (SDH). The forest plot shows k studies using the random effects model (first author, odds ratio, confidence interval, and weights). Information on the P-curve and GOSH plot was not displayed in panel c due to a reduced number of studies available for conducting specific analyses. The random effects model results (Demographics: k = 9, n = 99120, OR = 1.1232, p-value = 0.5132, CI = [0.7593; 1.6613]; Health: k = 8, n = 98992, OR = 1.4634, p-value = 0.1264, CI = [0.8708; 2.4595]; Mental health symptoms: k = 2, n = 49257, OR = 1.083, p-value = 0.0577, CI = [0.9878; 1.874]; Lifestyle: k = 4, n = 80130, OR = 1.3086, p-value = 0.2756, CI = [0.6875; 2.4906]; SDH: k = 8, n = 98793, OR = 1.2273, p-value = 0.1144, CI = [0.9382; 1.6055]) are reported with Knapp-Hartung correction for false discovery rate, the prediction interval, and heterogeneity values (I², tau²). Contour-enhanced funnel plot showing effect sizes, standard errors, and significance. P-curve analysis, showing the accumulation of p-values over the significant studies (observed p-curve), the no-effect curve, and the 33% power curve. GOSH (Graphic Display of Heterogeneity) shows the distribution for all 2k-1 possible study combinations (1 million randomly selected models when 2k-1 > 106) in blue and leaving out the most negatively influential study in green. Blank panels indicate that P-Curves and GOSH cannot be estimated due to insufficient studies.

A meta-analysis on the demographic factors associated with functional ability (age and sex, *k* = 9) did not reach significance (OR = 1.1232, *P* = 0.5132, CI = (0.7593, 1.6613)). High heterogeneity was detected (*I*^2^ = 95.9%, CI = (93.4%, 97.1%)). The pooled effect size without outliers presented high heterogeneity (*I*^2^ = 94.8%, CI = (91.6%, 96.8%)) and non-statistical significance (OR = 1.137, *P* = 0.2887, CI = (0.8681, 1.4891); Table 3, Extended Data Fig. 2a and Supplementary Figs. 1–3). Subgroup analysis showed significant differences between countries (*P* < 0.0001), and effect sizes ranged from 0.6633 to 2.5641 (Extended Data Table 4). Egger’s test (*P* = 0.5791) did not indicate asymmetry (Extended Data Table 5 and Extended Data Fig. 2a), and p-curve analysis showed significant results for the half and full p-curve tests, indicating the presence of a true non-zero effect (z full = −11.177, *P* < 0.001, z half = −12.303, *P* < 0.001, power estimate = 99%, CI = (99%, 99%); Extended Data Table 6 and Extended Data Fig. 2a).

#### Health status

A meta-analysis on the health factors associated with functional ability (cardiometabolic diseases and other, *k* = 8) did not reach significance (OR = 1.4634, *P* = 0.1264, 95% CI = (0.8708, 2.4595)) and exhibited high heterogeneity (*I*^2^ = 79.6%, CI = (60.2%, 89.5%)). After excluding outliers, heterogeneity decreased, although it remained high (*I*^2^ = 58.5%, CI = (4.3%, 82%)). Concurrently, the pooled effect size exhibited statistical significance with narrower CIs (OR = 1.1921, *P* < 0.0003, 95% CI = (1.1260, 1.2622); Table 3, Extended Data Fig. 2b and Supplementary Figs. 1–3). Subgroup analysis showed significant country differences (*P* = 0.0288), and effect sizes ranged from 0.9227 to 1.9227 (Extended Data Table 1). Egger’s test (*P* = 0.0926) did not indicate asymmetry (Extended Data Table 2 and Extended Data Fig. 2c). p-curve analysis showed significant results for half and full p-curve tests, indicating the presence of a true non-zero effect (z full = −11.004, *P* < 0.001, z half = –10.804, *P* < 0.001, power estimate = 96%, CI = (83.1%, 99%); Extended Data Table 3 and Extended Data Fig. 2b).

#### Mental health symptoms

A meta-analysis on the role of mental health symptoms on functional ability (depression, anxiety and other, *k* = 2) did not show significant effects (OR = 1.083, *P* = 0.0577, CI = (0.9878, 1.874)). The *I*^2^ and other statistics were not calculated due to insufficient studies (Extended Data Table 4 and Extended Data Fig. 2c).

#### Lifestyle

The lifestyle factors meta-analysis (physical activity, nutrition, alcohol consumption and smoking, *k* = 4) did not reach significance (OR = 1.3086, *P* = 0.2756, 95% CI = (0.6875, 2.4906)) and showed high heterogeneity (*I*^2^ = 99.4%, CI = (99.2%, 99.6%)). No outliers were detected (Extended Data Table 4, Extended Data Fig. 2d and Supplementary Figs. 1–3). Subgroup analysis did not show significant country differences (*P* = 0.7242), and effect sizes ranged from 1.1597 to 1.9564 (Extended Data Table 4). Egger’s test (*P* = 0.3858) did not indicate asymmetry (Extended Data Table 5 and Extended Data Fig. 2d). p-curve analysis showed significant results for both half and full p-curve tests, indicating the presence of a true non-zero effect (z full = −12.412, *P* < 0.001, z half = −12.246, *P* < 0.001, power estimate = 99%, CI = (97.9%, 99%); Extended Data Table 6 and Extended Data Fig. 2d).

#### SDH

The SDH factors meta-analysis (educational attainment and socioeconomic status, *k* = 8) did not reach significant effects on functional ability (OR = 1.2273, *P* = 0.1144, 95% CI = (0.9382, 1.6055)) and showed high heterogeneity (*I*^2^ = 99%, CI = (98.7%, 99.2%)). After excluding outliers, the random effects model showed a statistically significant effect (OR = 1.3401, *P* = 0.0328, CI = (1.0338, 1.7371)), and heterogeneity remained high (*I*^2^ = 99.1%, CI = (98.8%, 99.3%); Extended Data Table 4, Extended Data Fig. 2e and Supplementary Figs. 1–3). Subgroup analysis did not show significant differences between countries (*P* = 0.7294), and effect sizes ranged from 1.0586 to 1.3086 (Extended Data Table 4). Egger’s test (*P* = 0.8271) did not indicate asymmetry (Extended Data Table 5 and Extended Data Fig. 2e). p-curve analysis showed significant results for both half and full p-curve tests, indicating the presence of a true non-zero effect (z full = −10.614, *P* < 0.001, z half = −10.099, *P* < 0.001, power estimate = 99%, CI = (99%, 99%); Extended Data Table 6 and Extended Data Fig. 2e).

### Scoping review results

A complete list of studies included in all analyses for cognition^1^^,23–49^ and functional ability^1,28,50–57^ is included here and further described in Supplementary Table 1. This table includes the review, citations, sample sizes, methods employed, effect sizes, results and methodological gaps identified. Most of the studies (96%) did not use data-driven approaches to avoid a priori bias in the selection of predictors from other regions. Similarly, most of the studies (94%) involved a single country from Latin America with no country comparisons; used no machine learning with standard procedures to assess overfitting and generalization; and did not combine cross-sectional and longitudinal studies, nor did they use any multi-method complementary analysis for confirmation. In most of the studies (92%), there was a lack of training and test partition, and they relied on a single data source for training and testing. Similarly, most of the studies did not use heterogeneity-robust methodologies (80%). Also, some of these studies focused on patients without considering healthy aging (32%) and did not assess multiple potential predictors (28%).

## Discussion

The meta-analyses and review reveal significant heterogeneity in the relationship between various risk factors and both cognition and functional ability in Latin America. In high-income countries, meta-analyses and systematic reviews have demonstrated associations between healthy aging and most of the factors addressed in this work^11–13,15,16^. The identification of underlying true non-zero effects across many analyses suggests that these areas are rich grounds for further research. However, the presence of high heterogeneity, the impact of outliers and publication bias all stress weakness of the available evidence. The review of methodological procedures indicates multiple flaws in the literature. Thus, our results undermine the reliability and generalizability of the available research, limiting the ability to develop tailored prevention and intervention programs for healthy aging in the region.

Considering all factors, the meta-analyses showed true non-zero effects. For cognition, robust effects were observed only after excluding outliers. The heterogeneity and significant differences between countries point to inconsistent effects^9,10,20,19^. For functional ability, small but significant effects were observed but with narrow CIs after excluding outliers. A minor number of reports showed stable effects, contrasting with other meta-analyses in the United States and Europe^11–16^. Overall, although significantly influencing aging, the main risk factors of healthy aging exhibit large heterogeneity and lack specificity.

The effects of specific factors were even less robust. Demographics significantly influence cognition, albeit with substantial heterogeneity and publication bias. For functional ability, the evidence was weaker, although an underlying true non-zero effect exists. An association between health status and both cognition and functional ability was demonstrated but with high heterogeneity. Mental health symptoms influence cognition, with significant heterogeneity and potential publication bias, and non-significant effects were observed on functional ability. For both cognition and functional ability, the primary analyses for SDH were non-significant. However, p-curve analyses indicated an underlying true non-zero effect, suggesting a minor effect. Conservatively, ranking the effects by CI without outliers yields demographics > mental health > health status effects, with lifestyle and SDH being not significant. This sequence contradicts previous reports^1^. Lifestyle and SDH were less frequently assessed, and measures varied across studies. Just a few studies evidenced significant effects of SDH. Most of the reports used disparate measures to track SDH. When studies include a few countries with harmonized measures, significant effects arise^1^. The lack of systematization in SDH across the region may lessen their importance as predictors. Additionally, an intrinsic heterogeneity of SDH is present within LACs^1^^,66,67^. This variation arises from differences in socioeconomic development, healthcare infrastructure, education access, ethnic and racial diversity and public health initiatives across countries, among other factors^66,67^. Consequently, the inherent diversity within LACs and the challenges in measuring regional social risks could skew our understanding of SDH in aging. The prediction of functional ability was weaker than cognition, likely due to fewer studies and less direct and objective assessment methods^1^. Overall, the evidence regarding specific factors associated with healthy aging appears inconclusive.

Some methodological factors extended the last conclusion. To address outliers, we implemented a procedure designed to reduce heterogeneity. The significance of the procedure lies in the fact that outliers often stem from atypical values, not from a broader distribution of values that indicate more significant inequalities. However, despite excluding outliers, significant heterogeneity persisted in most cases. This implies that high heterogeneity is a fundamental attribute of the datasets. The high *I*^2^ values showcase a considerable variation across studies, and exclusion of outliers was required to maintain data integrity, raising concerns about the representativeness of the findings^61^. Additionally, Egger’s test^58^ indicated asymmetry in some analyses, hinting at potential publication biases^59^. This test may lack statistical power to detect bias when the number of studies is small (*k* < 10), and, therefore, the results provided for functional ability must be read with caution. Different statistical constrains, including Knapp–Hatung adjustments^65^ and the Paule–Mandel estimator^64^, were used to try to reduce false discovery rates. Although theoretically sound, these measures might add layers of complexity and potential sources of error.

The scoping review provided additional caveats. Considerable research was concentrated on individual nations, neglecting the opportunity to address the regional population dynamic. Moreover, a lack of data-driven approaches that can avoid predefined biases from other regions constitutes a substantial barrier to the development of representative insights. With some exceptions, limited applications of machine learning techniques with data-driven feature selection, *k*-fold cross-validation and out-of-sample testing^68^ were used in the revised studies. These methods are instrumental in overcoming methodological biases associated with assuming a priori theoretical hierarchies in studying factors associated with clinical outcomes from other populations that are not necessarily equivalent^1,5,22^. Furthermore, these methods can better assess complex interactions between multiple variables related to an outcome while controlling for overfitting and ensuring out-of-sample validation^68^. However, machine learning algorithms are not free of other biases regarding the type of variables, missing data and contextual interpretation^69^. To determine unbiased predictors, non-data-driven domain expertise is important in aging research, and tailored programs would require causal methodology^69^. Combining cross-sectional and longitudinal studies can introduce biases related to survival and attrition. Longitudinal studies might overrepresent some positive factors associated with healthy aging, as they often include only older individuals who demonstrate high survival outcomes. These differences can profoundly affect the interpretation of results, potentially leading to a skewed representation of health outcomes. However, due to the unbalanced nature and scarcity of studies in this field, conducting sufficiently robust separate analyses is challenging. In any case, the currently available data in the region must be interpreted with caution. These results point to the necessity for a critical reassessment of the current approaches^2,10,17^ and the development of a more robust, tailored and inclusive research landscape^69^.

Results underscore the urgent need for systematic assessments of geographical variation and improved harmonization. These improvements will enable us to distinguish between components related to the intrinsic variability of populations, influenced by diverse genetic–environmental interactions^18^, and those arising from methodological limitations in current datasets. The substantial impact of outliers and publication bias highlights the weaknesses in the available evidence. Furthermore, our review of methodological procedures has uncovered several shortcomings in the existing literature. These shortcomings include inconsistent methods for assessing risk factors, focusing on individual predictors rather than their interactions, inadequate harmonization in assessing predictors and outcomes related to healthy aging and reliance on weak predictive models. Additionally, most studies of aging predictors do not employ data-driven approaches, thus avoiding potential biases associated with including pre-defined theoretical categories from other regions^70^.

Our study has limitations that underscore the need for further research. Most of the reports assessed had relatively small sample sizes compared to other regions^11–16^. This was particularly evident in the analysis of functional ability and the use of specific predictors, such as lifestyle and SDH. Significant discrepancies in sample sizes among studies may have skewed results, with larger samples reducing statistical uncertainty and, therefore, having a greater influence on the pooled effect size determined by the random effects models. The use of non-harmonized measures for risk factors, combined with the absence of interactions among variables, might have increased heterogeneity and diminished the prominence of specific factors. Including dissimilar studies, exposures and outcomes in a meta-analysis could partially explain heterogeneity. However, other meta-analyses from other regions followed this approach and found consistent results^11–13,15,16^. The pooled effect might not faithfully represent the actual effect size, which could differ across populations, contexts or study designs. This limits the generalizability of our findings and their overall statistical significance. The observed heterogeneity could also be influenced by factors not covered in the studies, such as genetics, population admixture, healthcare infrastructure and others^1,2,9,10,17,19–21^. Some reports have highlighted the premature mortality in LACs^71^, associated with socioeconomic disparities^71^. However, there is contrasting information when compared to populations in the United States. A lower rate of premature mortality among both male and female Latinos living in the United States has been called ‘the Latin American paradox’^72^. Future studies should adjust for regional mortality to better reflect the unique sociodemographic dynamics in the region. Addressing the effects according to World Bank categorization would provide useful information. However, when segmenting by domain and income, an unbalanced and small number of studies are observed in several categories, hindering the feasibility of such analyses. Similarly, risk factors in high-income countries within Latin America do not follow standard trends according to countries’ income seen in other regions^5,22^. Moreover, our search and meta-analysis analyzed various studies, including population-representative research, clinical samplings of participants visiting clinics or healthcare providers and community-dwelling studies (Supplementary Table 1). The diversity of these studies could partially explain the heterogeneity effects observed in the meta-analysis. Future research should control for the type and source of participants to assess the impact of risk factors on healthy aging across Latin America. Finally, although our scoping review focused on methodological gaps, future reviews should consider other potential aspects.

In conclusion, our results indicate a lack of robust healthy aging risk effects in Latin America, highlighting the need for more studies and better-quality research methods. The inherent limitations of current databases in LACs, along with the urgent need for harmonized assessment protocols, highlight the critical need for governments, stakeholders and researchers to collaborate in supporting the development of open, shareable, harmonized and multicentric data science initiatives dedicated to aging research in the region. The observed large heterogeneous effects, disparate results across countries, small effect sizes and publication bias all emphasize the urgent need for more systematic research. The inherent limitations of current databases in LACs, along with the urgent need for harmonized assessment protocols, highlight the critical need for governments, stakeholders and researchers to collaborate in supporting the development of open, shareable, harmonized and multicentric data science initiatives dedicated to aging research in the region. The findings advocate for developing more research to promptly address healthy aging and its multiple risk factors in Latin America, enabling governments to craft truly evidence-based initiatives for prevention and intervention.

## Methods

### Search and selection criteria

In this meta-analysis and review, we searched MEDLINE, PsycINFO and Virtual Health Library databases for studies published between the database inception and 30 March 2023. We contacted the corresponding authors to obtain the required data when they were not reported. Studies were excluded if the required data were not obtained after at least two attempts. Figure 1 shows the study design. We used a combined set of keywords related to aging, SDH, health status, mental health symptoms, lifestyle, demographics and Latin America (Supplementary File 1) to identify human studies reporting factors associated with cognition and functional ability in the region (Fig. 2). We also checked the reference lists of the retrieved studies for relevant reports that could be included in the current review. Randomized controlled trials, cohort studies and cross-sectional studies that met the eligibility criteria were included. Studies had to be (1) published in a peer-reviewed journal, (2) written in English, Spanish, Portuguese or French and (3) reporting on data collected from humans. A set of 38 studies was used for both the scoping review and the meta-analysis. These comprised 28 studies on cognition and 10 on functional ability. Independent assessments were conducted by reviewers for both the scoping review and the meta-analysis. We adhered to PRISMA guidelines for scoping reviews, ensuring that our methodology was transparent and replicable. Our study did not include randomized clinical trials or case–control studies, as we focused on identifying factors associated with healthy aging instead of those linked to aging-related diseases.

### Ethics and inclusion statement

This work involved a collaboration among scientists in multiple countries, including Argentina, Chile, Colombia and Ireland. Contributors from all sites are included as coauthors or in acknowledgements according to their contributions. Researchers residing in LACs were involved in study design, study implementation, methodological procedure and writing and reviewing processes. Roles and responsibilities were agreed among collaborators ahead of the research. Local ethics committees of each database approved this research. To prevent any stigmatization, all identifying information was removed to preserve the privacy of individuals. Each country included in this study retained ownership of all human material shared for research purposes. We endorse the Nature Portfolio journals’ guidance on low- and middle-income country (LMIC) authorship and inclusion. Authorship was based on the intellectual contribution, commitment and involvement of each researcher in this study. We included authors born in LMICs and other underrepresented countries in this study. This study holds local relevance for each investigated country by presenting disaggregated findings, thereby offering country-specific risk factors of healthy aging. The selection of variables was informed by previous research and in accordance with established guidelines for global aging studies.

### Meta-analysis

#### Clinical outcomes

Different meta-analyses were run for risk factors of cognition and functional ability in Latin American populations. A list of variables used to track each outcome is provided in Supplementary Table 2. For each outcome (cognition and functional ability), we ran six meta-analyses, including a global meta-analysis studying all risk factors and five independent meta-analyses for each factor studied independently. Summarized results can be found in Tables 1, 2 and 3.

#### Risk factors

Due to the significant heterogeneity of the predictors observed by the studies, they were grouped into five categories: demographics (age and sex), health (cardiometabolic risks and other somatic diseases), mental health symptoms (depression and anxiety), lifestyle (physical activity, nutritional habits, alcohol consumption and smoking) and SDH (educational attainment and socioeconomic status). Educational attainment refers to the highest level of education that an individual has completed. This can include levels such as a high school diploma, vocational training, undergraduate degrees or advanced degrees such as master’s or doctoral degrees. Some studies assessed education by determining the years of completed formal education^1^. This work includes studies that assessed education using education attainment measures and years of formal education. Socioeconomic status refers to the individual’s or group’s level in society, typically determined by a combination of factors, including educational background, income level and occupation^73^. A list of variables used to track educational attainment and socioeconomic status is provided in Supplementary Table 3. Other variables, such as ethnicity, health access and social adversities, were not included in further analyses because few studies incorporated these measures, and there was no systematic or reproducible approach across the studies.

#### Data analysis

A group of three reviewers extracted information on authors, year of publication, effect size, CI, type of effect size, predictors, outcomes, population, country study design and analyses and control variables of all articles. These were verified for accuracy by another independent reviewer. Any missing fields were left blank. For assessing publication bias, we used Egger’s test^58^ and enhanced funnel plots^59^. Additionally, we assessed the strength and possible bias of the empirical evidence we found as source of this meta-analysis with the p-curve test^60^. However, this approach is not robust when high heterogeneity exists between studies. For this reason, we also included the *I*^2^ index^61^, a measure of the proportion of the estimates of variance due to heterogeneity. This metric is independent of the number of studies and can be compared across meta-analyses with a different number of studies and metrics. Values above 35% are considered as reflecting high heterogeneity in the group of studies assessed. Heterogeneity was further explored with *I*^2^ GOSH plots^62^. To explore heterogeneity even further, subgroup analyses were performed between countries. When outlying effect sizes were identified based on previous criteria, we conducted subsequent analyses by pooling effects, excluding outliers^63^.

We employed random effects models to analyze all combined factors as well as each factor individually. The Paule–Mendel estimator was used, given its suitability for scenarios with a limited number of studies. Furthermore, we incorporated Knapp–Hartung adjustments to account for false discovery^65^. Only one article did not report adequate statistics (logistic regression models), and we transformed the reported Cohen’s *f* for every model using Cohen’s *d* transformations to log OR using the following formula^74^:

$${{\rm{ln(OR)}}}=\frac{d\times\pi }{\sqrt{3}}$$

For each model in this study, we first converted Cohen’s *f* to Cohen’s *d* (*f* = *d*/2; ref. ^75^). Afterwards, we converted it to the log OR^74^ (log OR = *d* × phi/sqrt). This conversion allowed us to achieve a consistent analytical framework across studies.

Following previous classifications^76^, we define an outlier when the CIs do not overlap with those of the pooled effect size, indicating that its effect size is extreme and differs significantly from the overall effect^76^. All analyses were conducted in R (version 4.1.2) using additional packages specifically developed for meta-analyses.

Our approach involves harmonizing the values of predictors and their effects on outcomes^74,76^ by converting them into ORs and additional steps. We identified the most analogous variables across each study and incorporated those variables into our analyses, following established procedures^74,76^ and previous meta-analyses^77–79^. This enabled us to obtain specific ORs for each factor in every study and transform their effect sizes^74,76^, making the different variables comparable. We also provided the individual effects at the study level, highlighting the unique effects. Such procedures allowed us to assess the heterogeneity across studies, achieving partial harmonization and providing individual report metrics. However, the inherent limitations of current databases in LACs, along with the urgent need for harmonized assessment protocols, highlight the critical need for governments, stakeholders and researchers to collaborate in supporting the development of open, shareable, harmonized and multicentric data science initiatives dedicated to aging research in the region. All models and statistical analyses were run using Python version 3.9.13.

### Scoping review on methodological approaches

Following the criteria detailed in the search and selection section (Supplementary File 1), we initially identified 455 studies. After a detailed review of the titles and abstracts, we narrowed down to 38 studies that fully met the criteria for our proposed scoping review. We analyzed several methodological aspects across studies, including (1) evaluating simultaneous associations and the interplay between various potential factors that could be related to healthy aging; (2) determining whether the studies used cross-sectional, longitudinal or combined methods; (3) analyzing the use of data-driven approaches to mitigate potential biases arising from theoretical assumptions; (4) assessing the representation of countries with different socioeconomic backgrounds; and (5) examining the deployment of robust methodological approaches for predictions, which included measures to control overfitting, out-of-sample validation, cross-validation and other adequate standards of analysis. This approach ensured a comprehensive and thorough analysis, facilitating a robust review grounded in stringent criteria that allowed for the inclusion of a variety of perspectives and methodologies.

### Statistics and reproducibility

In this meta-analysis and scoping review, no statistical method was used to pre-determine sample size. No data were excluded from the analyses. The procedures implemented for meta-analysis and scoping review followed international guidelines. The investigators assessed independently the group of studies to the meta-analysis and the scoping review. No experimental or blinded procedures were used in this study.

### Reporting summary

Further information on research design is available in the Nature Portfolio Reporting Summary linked to this article.

### Supplementary information

Supplementary File 1 and Figs. 1–3.

Reporting Summary

Supplementary Tables 1–3. Table 1. Main results of studies assessing risk factors of functional ability and cognition. Table 2. Measures used to assess cognition and functionality across studies. Table 3. Measures used to track social determinants of health and socioeconomic disparities across studies.

## Figures and Tables

**Extended Data Table 1:**
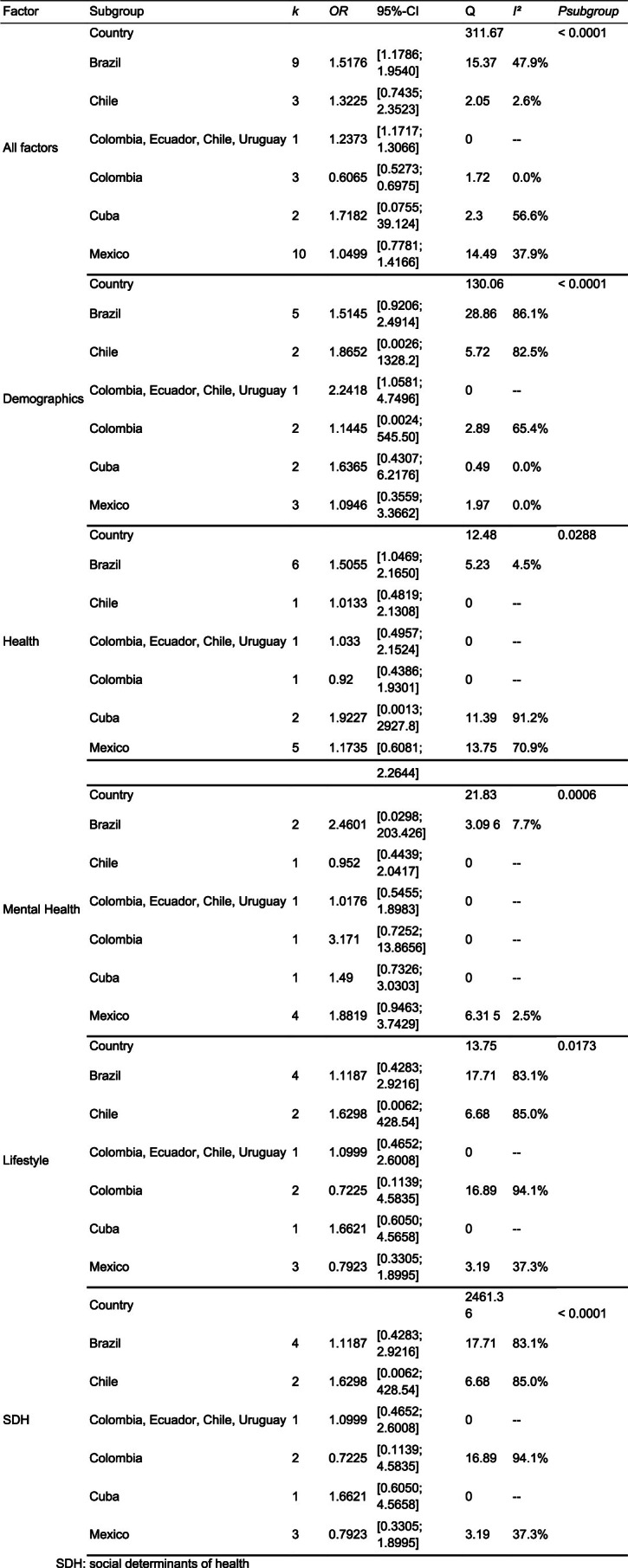
Meta-analyses of cognition by country

**Extended Data Table 2:**
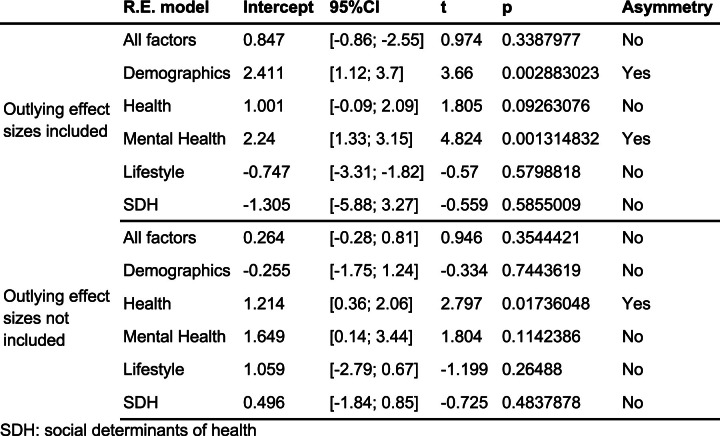
Publication bias test (Egger’s test) of cognition

**Extended Data Table 3:**
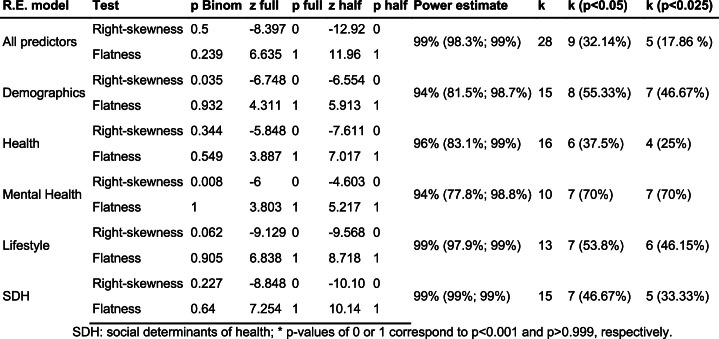
Analyses of p-curve of cognition

**Extended Data Table 4:**
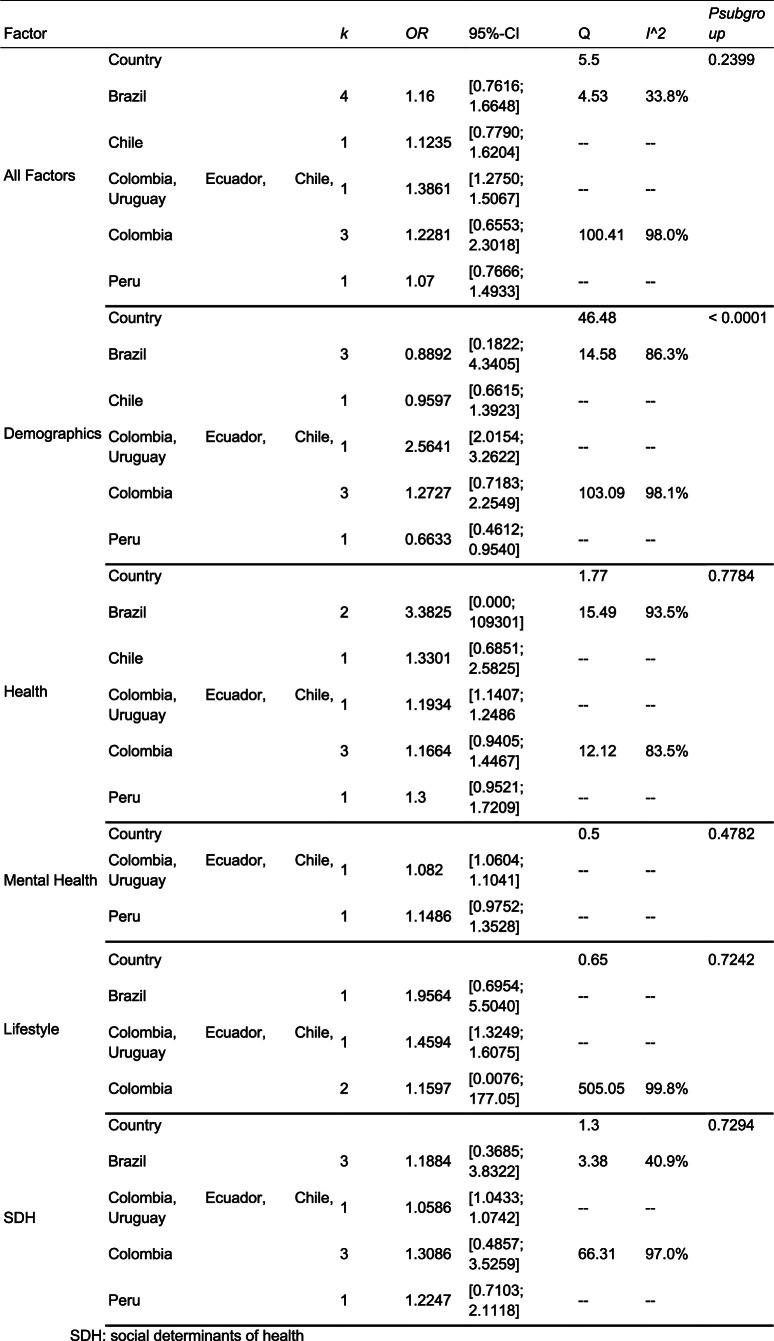
Meta-analyses of functional ability by country

**Extended Data Table 5:**
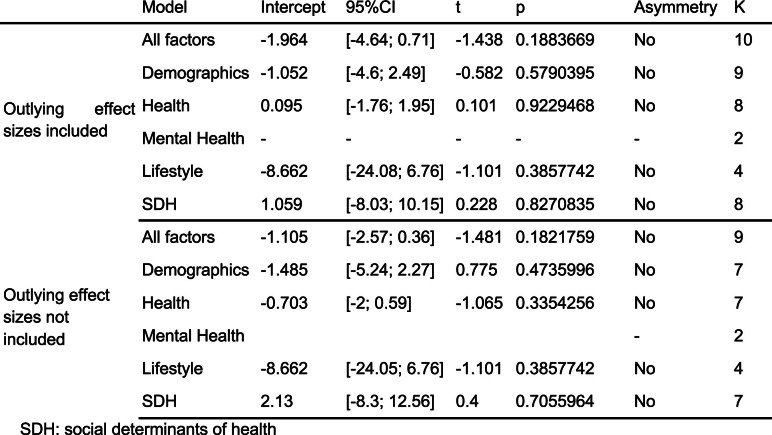
Publication bias test (Egger’s test) of functional ability

**Extended Data Table 6:**
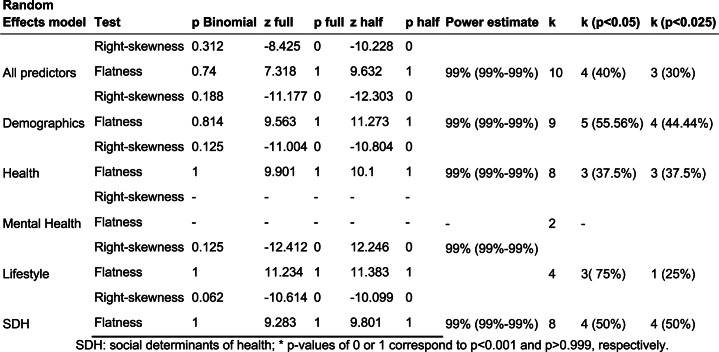
Analyses of p-curve in functional ability

## Data Availability

Data included in this study are available on GitHub at https://github.com/AI-BrainLat-team/Latam-Aging-Meta-Analysis/tree/main/Data. Data from studies analyzed in the meta-analysis and scoping review are summarized in the Results section. Furthermore, all data are reported in Supplementary Table 1 and Supplementary File 1.

## Code availability

All code for data analysis associated with this manuscript is available for download on GitHub at https://github.com/AI-BrainLat-team/Latam-Aging-Meta-Analysis.
