# Supplementary material for: Healthy aging meta-analyses and scoping review of risk factors across Latin America reveal large heterogeneity and weak predictive models
Source: Nat Aging. 2024 Jun 17;4(8):1153–65. doi: 10.1038/s43587-024-00648-6 (PMC11333291; doi:10.1038/s43587-024-00648-6)
Supplement: Supplementary file 1 — and Figs. 1–3. [file 43587_2024_648_MOESM1_ESM.pdf]

nature aging

Supplementary information

https://doi.org/10.1038/s43587-024-00648-6

Healthy aging meta-analyses and scoping review of risk factors across Latin America reveal large heterogeneity and weak predictive models

In the format provided by the authors and unedited

Supplementary file 1. Search strategy (16/8/2023)

MEDLINE: 313

("brain health"[All Fields] OR "brain health 120 dementia"[All Fields] OR "healthy aging"[All Fields] OR "cognitive health"[All Fields] OR "cognitive health and aging"[All Fields] OR "aging brain health"[All Fields] OR "instrumental functioning"[All Fields] OR "instrumental functionality"[All Fields] OR "aging health"[All Fields] OR "health functioning"[All Fields] OR "daily life functioning"[All Fields] OR "barthel index"[All Fields] OR "lawton"[All Fields] OR "minimental state examination"[All Fields] OR "mmse"[All Fields] OR "cognitive screening"[All Fields] OR "dementia"[All Fields] OR "cognitive decline"[All Fields] OR "mild cognitive impairment"[All Fields] OR "cognitive impairment"[All Fields] OR "functional ability"[All Fields] OR "mild behavior impairment"[All Fields] OR "mild behavioral impairment"[All Fields] OR "mild behavioral impairment checklist"[All Fields] OR "neuropsychiatric inventory"[All Fields] OR "functional decline"[All Fields] OR "daily life disturbance"[All Fields] OR "instrumental functioning"[All Fields] OR "instrumental functionality"[All Fields] OR "instrumental functioning"[All Fields] OR "cognition"[All Fields] OR "cognitive functioning"[All Fields] OR "cognitive screening"[All Fields] OR "neurocognitive disorders"[All Fields]) AND ("prediction"[All Fields] OR "predictor"[All Fields] OR "risk factor"[All Fields] OR "protective factor"[All Fields] OR "preventable factors"[All Fields] OR "social determinants of health"[All Fields] OR "socioeconomic status"[All Fields] OR "education level"[All Fields] OR "scholarly"[All Fields] OR "scholarship"[All Fields] OR "years of education"[All Fields] OR "isolation"[All Fields] OR "social participation"[All Fields] OR "living alone"[All Fields] OR "social exclusion"[All Fields] OR "social group"[All Fields] OR "income"[All Fields] OR "inequalities"[All Fields], "inequities"[All Fields] OR "poverty"[All Fields] OR "health access"[All Fields] OR "neighborhood conditions"[All Fields] OR "age"[All Fields] OR "aging"[All Fields] OR "ageing"[All Fields] OR "female"[All Fields] OR "male"[All Fields] OR "women"[All Fields] OR "woman"[All Fields] OR "men"[All Fields] OR "man"[All Fields] OR "gender"[All Fields] OR "sex"[All Fields] OR "hypoacusia"[All Fields] OR "cardiometabolic risk"[All Fields] OR "cardiometabolic factors"[All Fields] OR "hypertension"[All Fields] OR "diabetes"[All Fields] OR "obesity"[All Fields] OR "gait speed"[All Fields] OR "handgrip strength"[All Fields] OR "heart disease"[All Fields] OR "cardiovascular risk"[All Fields] OR "cardiovascular disease"[All Fields] OR "stroke"[All Fields] OR "heart attack"[All Fields] OR "sedentarism"[All Fields] OR "physical activity"[All Fields] OR "smoking"[All Fields] OR "alcohol consumption"[All Fields] OR "nutrition habits"[All Fields] OR "nutritional factors"[All Fields] OR "lifestyle"[All Fields] OR "nutrition"[All Fields] OR "diet"[All Fields] OR "selfperceptions"[All Fields] OR "depression"[All Fields] OR "anxiety"[All Fields] OR "depressive symptoms"[All Fields] OR "anxiety symptoms"[All Fields] OR "depressive problems"[All Fields] OR "stress"[All Fields] OR "stress related disorders"[All Fields] OR "mental health"[All Fields] OR "quality of life"[All Fields]) AND (aruba OR bahamas OR barbados OR "cayman islands" OR cuba OR dominica OR "dominican republic" OR grenada OR guadeloupe OR haiti OR jamaica OR martinique OR 'puerto rico' OR "saint barthélemy" OR "virgin islands" OR belize OR "costa rica" OR 'el salvador' OR guatemala OR honduras OR mexico OR nicaragua OR panama OR argentina OR bolivia OR brazil OR chile OR colombia OR ecuador OR "french guiana" OR guyana OR paraguay OR peru OR suriname OR uruguay OR venezuela OR "South America" OR "Latin America" OR "Central America" OR "Hispanic Americans") AND ("machine learning methods"[All Fields] OR "random forest"[All Fields] OR "elastic net"[All Fields] OR "lasso"[All Fields] OR "logistic regression"[All Fields] OR "predictive model"[All Fields] OR "datadriven"[All Fields] OR "automatized analysis"[All Fields]) AND ("cross-sectional study" OR "National aging survey" OR "national survey"[All Fields] OR cross-sectional studies OR "longitudinal studies"[All Fields] OR "observational studies"[All Fields] OR "cohort studies"[All Fields] OR "10 66 cohort study"[All Fields] OR "sabe survey"[All Fields] OR "Encuesta de salud, bienestar y envejecimiento")

PsycNet: 132

(Any Field: "brain health" OR Any Field: "brain health 120 dementia" OR Any Field: "healthy aging" OR Any Field: "cognitive health" OR Any Field: "cognitive health and aging" OR Any Field: "aging brain health" OR Any Field: "instrumental functioning" OR Any Field: "instrumental functionality" OR Any Field: "aging health" OR Any Field: "health functioning" OR Any Field: "daily life functioning" OR Any Field: "barthel index" OR Any Field: "lawton" OR Any Field: "minimental state examination" OR Any Field: "mmse" OR Any Field: "cognitive screening" OR Any Field: "dementia" OR Any Field: "cognitive decline" OR Any Field: "mild cognitive impairment" OR Any Field: "cognitive impairment" OR Any Field: "functional ability" OR Any Field: "mild behavior impairment" OR Any Field: "mild behavioral impairment" OR Any Field: "mild behavioral impairment checklist" OR Any Field: "neuropsychiatric inventory" OR Any Field: "functional decline" OR Any Field: "daily life disturbance" OR Any Field: "instrumental functioning" OR Any Field: "instrumental functionality" OR Any Field: "instrumental functioning" OR Any Field: "cognition" OR Any Field: "cognitive functioning" OR Any Field: "cognitive screening" OR Any Field: "neurocognitive disorders") AND (Any Field: "prediction" OR Any Field: "predictor" OR Any Field: "risk factor" OR Any Field: "protective factor" OR Any Field: "preventable factors" OR Any Field: "social determinants of health" OR Any Field: "socioeconomic status" OR Any Field: "education level" OR Any Field: "scholarly" OR Any Field: "scholarship" OR Any Field: "years of education" OR Any Field: "isolation" OR Any Field: "social participation" OR Any Field: "living alone" OR Any Field: "social exclusion" OR Any Field: "social group" OR Any Field: "income" OR Any Field: "inequalities" OR Any Field: "inequities" OR Any Field: "poverty" OR Any Field: "health access" OR Any Field: "neighborhood conditions" OR Any Field: "age" OR Any Field: "aging" OR Any Field: "ageing" OR Any Field: "female" OR Any Field: "male" OR Any Field: "women" OR Any Field: "woman" OR Any Field: "men" OR Any Field: "man" OR Any Field: "gender" OR Any Field: "sex" OR Any Field: "hypoacusia" OR Any Field: "cardiometabolic risk" OR Any Field: "cardiometabolic factors" OR Any Field: "hypertension" OR Any Field: "diabetes" OR Any Field: "obesity" OR Any Field: "gait speed" OR Any Field: "handgrip strength" OR Any Field: "heart disease" OR Any Field: "cardiovascular risk" OR Any Field: "cardiovascular disease" OR Any Field: "stroke" OR Any Field: "heart attack" OR Any Field: "sedentarism" OR Any Field: "physical activity" OR Any Field: "smoking" OR Any Field: "alcohol consumption" OR Any Field: "nutrition habits" OR Any Field: "nutritional factors" OR Any Field: "lifestyle" OR Any Field: "nutrition" OR Any Field: "diet" OR Any Field: "selfperceptions" OR Any Field: "depression" OR Any Field: "anxiety" OR Any Field: "depressive symptoms" OR Any Field: "anxiety symptoms" OR Any Field: "depressive problems" OR Any Field: "stress" OR Any Field: "stress related disorders" OR Any Field: "mental health" OR Any Field: "quality of life") AND (Any Field: aruba OR Any Field: bahamas OR Any Field: barbados OR Any Field: "cayman islands" OR Any Field: cuba OR Any Field: dominica OR Any Field: "dominican republic" OR Any Field: grenada OR Any Field: guadeloupe OR Any Field: haiti OR Any Field: jamaica OR Any Field: martinique OR Any Field: 'puerto rico' OR Any Field: "saint barthélemy" OR Any Field: "virgin islands" OR Any Field: belize OR Any Field: "costa rica" OR Any Field: 'el salvador' OR Any Field: guatemala OR Any Field: honduras OR Any Field: mexico OR Any Field: nicaragua OR Any Field: panama OR Any Field: argentina OR Any Field: bolivia OR Any Field:

brazil OR Any Field: chile OR Any Field: colombia OR Any Field: ecuador OR Any Field: "french guiana" OR Any Field: guyana OR Any Field: paraguay OR Any Field: peru OR Any Field: suriname OR Any Field: uruguay OR Any Field: venezuela OR Any Field: "South America" OR Any Field: "Latin America" OR Any Field: "Central America" OR Any Field: "Hispanic Americans") AND (Any Field: "machine learning methods" OR Any Field: "random forest" OR Any Field: "elastic net" OR Any Field: "lasso" OR Any Field: "logistic regression" OR Any Field: "predictive model" OR Any Field: "datadriven" OR Any Field: "automatized analysis") AND Any Field: ("cross-sectional study" OR "National aging survey" OR "national survey" OR cross-sectional studies OR "longitudinal studies" OR "observational studies" OR "cohort studies" OR "10 66 cohort study" OR "sabe survey" OR “Encuesta de salud, bienestar y envejecimiento”)

Virtual health library: 8

("brain health" OR "brain health 120 dementia" OR "healthy aging" OR "cognitive health" OR "cognitive health and aging" OR "aging brain health" OR "instrumental functioning" OR "instrumental functionality" OR "aging health" OR "health functioning" OR "daily life functioning" OR "barthel index" OR "lawton" OR "minimental state examination" OR "mmse" OR "cognitive screening" OR "dementia" OR "cognitive decline" OR "mild cognitive impairment" OR "cognitive impairment" OR "functional ability" OR "mild behavior impairment" OR "mild behavioral impairment" OR "mild behavioral impairment checklist" OR "neuropsychiatric inventory" OR "functional decline" OR "daily life disturbance" OR "instrumental functioning" OR "instrumental functionality" OR "instrumental functioning" OR "cognition" OR "cognitive functioning" OR "cognitive screening" OR "neurocognitive disorders")) AND (("prediction" OR "predictor" OR "risk factor" OR "protective factor" OR "preventable factors" OR "social determinants of health" OR "socioeconomic status" OR "education level" OR "scholarly" OR "scholarship" OR "years of education" OR "isolation" OR "social participation" OR "living alone" OR "social exclusion" OR "social group" OR "income" OR "inequalities" OR "inequities" OR "poverty" OR "health access" OR "neighborhood conditions" OR "age" OR "aging" OR "ageing" OR "female" OR "male" OR "women" OR "woman" OR "men" OR "man" OR "gender" OR "sex" OR "hypoacusia" OR "cardiometabolic risk" OR "cardiometabolic factors" OR "hypertension" OR "diabetes" OR "obesity" OR "gait speed" OR "handgrip strength" OR "heart disease" OR "cardiovascular risk" OR "cardiovascular disease" OR "stroke" OR "heart attack" OR "sedentarism" OR "physical activity" OR "smoking" OR "alcohol consumption" OR "nutrition habits" OR "nutritional factors" OR "lifestyle" OR "nutrition" OR "diet" OR "selfperceptions" OR "depression" OR "anxiety" OR "depressive symptoms" OR "anxiety symptoms" OR "depressive problems" OR "stress" OR "stress related disorders" OR "mental health" OR "quality of life")) AND ((aruba OR bahamas OR barbados OR "cayman islands" OR cuba OR dominica OR "dominican republic" OR grenada OR guadeloupe OR haiti OR jamaica OR martinique OR 'puerto rico' OR "saint barthélemy" OR "virgin islands" OR belize OR "costa rica" OR 'el salvador' OR guatemala OR honduras OR mexico OR nicaragua OR panama OR argentina OR bolivia OR brazil OR chile OR colombia OR ecuador OR "french guiana" OR guyana OR paraguay OR peru OR suriname OR uruguay OR venezuela OR "South America" OR "Latin America" OR "Central America" OR "Hispanic Americans")) AND (("machine learning methods" OR "random forest" OR "elastic net" OR "lasso" OR "logistic regression" OR "predictive model" OR "datadriven" OR "automatized analysis")) AND (("cross-sectional study" OR "National aging survey" OR "national survey" OR cross-sectional studies OR "longitudinal studies" OR "observational studies" OR "cohort studies" OR "10 66 cohort study" OR "sabe survey" OR “Encuesta de salud, bienestar y envejecimiento”)

Web of Science: 2

ALL=("brain health" OR "healthy aging" OR "cognitive health" OR "cognitive health and aging" OR "aging brain health" OR "instrumental functioning" OR "instrumental functionality" OR "aging health" OR "health functioning" OR "daily life functioning" OR "Barthel index" OR "Lawton" OR "minimental state examination" OR "cognitive screening" OR "dementia" OR "cognitive decline" OR "mild cognitive impairment" OR "cognitive impairment" OR "functional ability" OR "mild behavior impairment" OR "mild behavioral impairment" OR "mild behavioral impairment checklist" OR "neuropsychiatric inventory" OR "functional decline" OR "daily life disturbance" OR "instrumental functioning" OR "instrumental functionality" OR "instrumental functioning" OR "cognition" OR "cognitive functioning" OR "cognitive screening" OR "neurocognitive disorders")

ALL=("hypoacusia" OR "cardiometabolic risk" OR "cardiometabolic factors" OR "hypertension" OR "diabetes" OR "obesity" OR "gait speed" OR "handgrip strength" OR "heart disease" OR "cardiovascular risk" OR "cardiovascular disease" OR "stroke" OR "heart attack" OR "sedentarism" OR "physical activity" OR "smoking" OR "alcohol consumption" OR "nutrition habits" OR "nutritional factors" OR "lifestyle" OR "nutrition" OR "diet" OR "selfperceptions" OR "depression" OR "anxiety" OR "depressive problems" OR "stress" OR "mental health" OR "quality of life" OR "predictor" OR "risk factor" OR "protective factor" OR "preventable factors" OR "social determinants of health" OR "socioeconomic status" OR "education level" OR "scholarly" OR "scholarship" OR "years of education" OR "isolation" OR "social participation" OR "living alone" OR "social exclusion" OR "social group" OR "income" OR "inequalities" OR "inequities" OR "poverty" OR "health access" OR "neighborhood conditions" OR "aging" OR "ageing" OR "gender" OR "sex")

ALL=("South America" OR "Latin America" OR "Central America" OR "Hispanic Americans")

ALL=("machine learning methods" OR "random forest" OR "elastic net" OR "lasso" OR "logistic regression" OR "predictive model" OR "datadriven" OR "automatized analysis")

ALL=("10 66 cohort study" OR "sabe survey" OR “Encuesta de salud, bienestar y envejecimiento”)

Supplementary figures

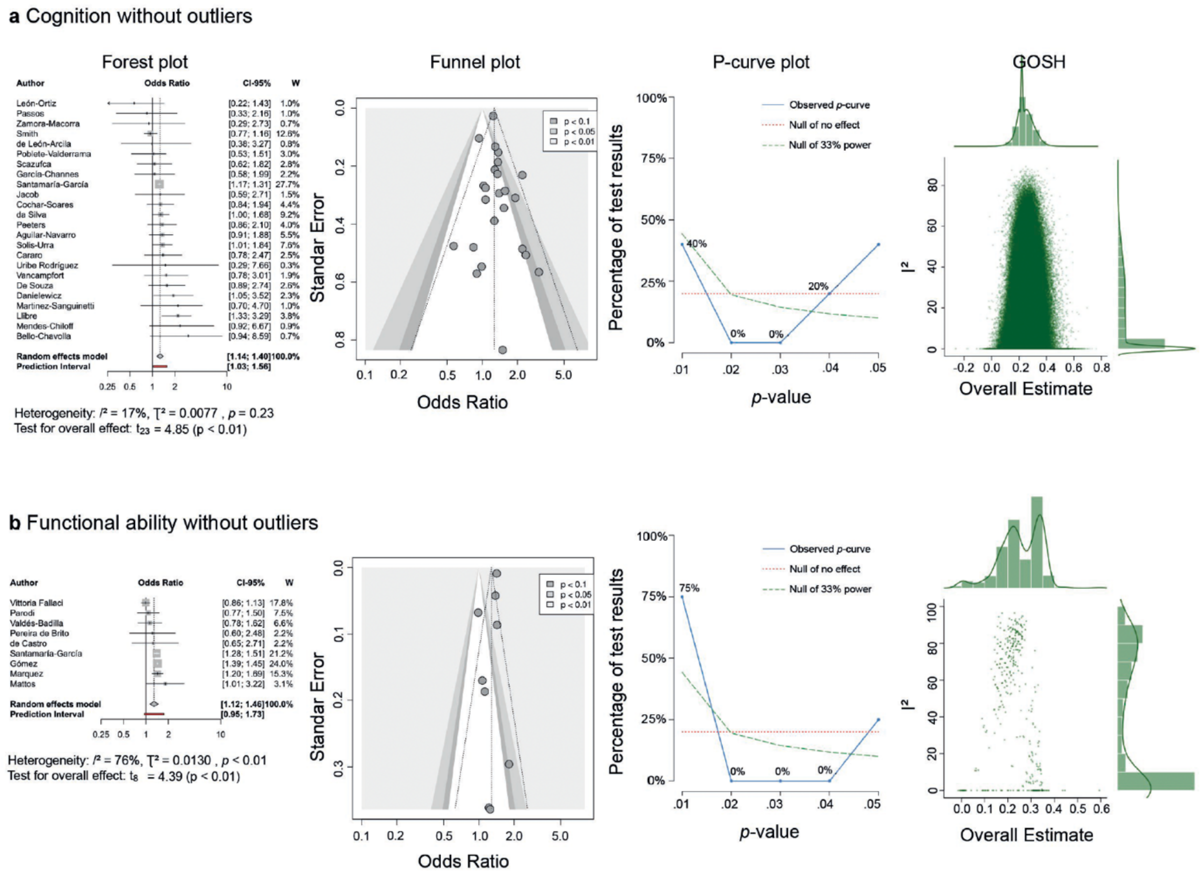

Supplementary Figure 1: Metanalysis of cognition and functional ability without outliers. Panel a shows results for cognition and **panel b** results for functional ability. Forest plot shows the studies in random effects model (first author, Odd Ratio, Confidence Interval, and weights). The random effects model results (Cognition: k = 24, n = 102064, OR = 1.2651, p-value = 0.00001, CI = [1.1443; 1.3987]; Functional ability: k = 9, n = 99428, OR = 1.2795, p-value = 0.0023, CI = [1.242; 1.4562]) are reported with Knapp-Hartung correction for false discovery rate, the prediction interval and heterogeneity values (I^2, tau^2). Contour-enhanced funnel plot showing effect sizes, standard errors, and significance. P-curve analysis, showing the accumulation of p-values over the significant studies (observed p-curve), the no-effect curve and 33% power curve. GOSH (Graphic Display of Heterogeneity) shows distribution for all 2^{k-1} possible study combinations (1 million randomly selected models when $2^{k-1} > 10^6$) in blue and leaving out the most negatively influential study in green.

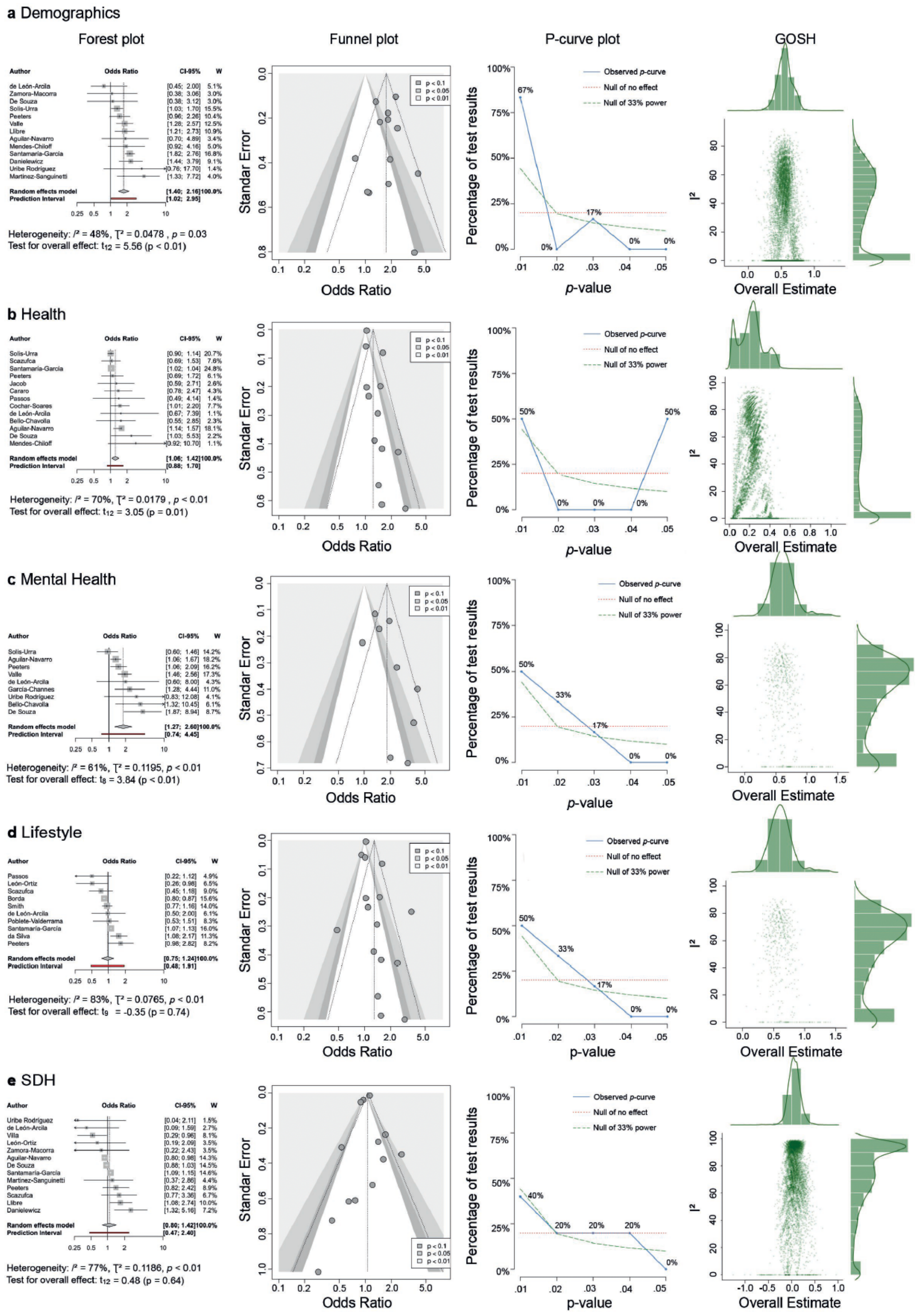

Supplementary Figure 2: Cognition without outliers. Each panel illustrates the major effects for each factor analyzed: **panel a** focuses on demographics; **panel b** on health; **panel c** on mental health; **panel d** on lifestyle; and **panel e** on Social Determinants of Health (SDH). Forest plot shows the studies in random effects model (first author, Odd Ratio, Confidence Interval, and weights). The random effects model results (Demographics: k = 13, n = 66634, OR = 1.73862, $p\text{-}value$ = 0.0001, CI = [1.3997;

2.1595]; Health: $k = 13$, $n = 67736$, $OR = 1.2259$, *p-value* = 0.00001, $CI = [1.0601; 1.4175]$; Mental health symptoms: $k = 9$, $n = 38679$, $OR = 1.8175$, *p-value* = 0.0049, $CI = [1.2699; 2.6012]$; Lifestyle: $k = 10$, $n = 89457$, $OR = 0.9623$, *p-value* = 0.7364, $CI = [0.7490; 1.2363]$; SDH: $k = 13$, $n = 68250$, $OR = 1.0658$, *p-value* = 0.6373, $CI = [0.7997; 1.4205]$) are reported with Knapp-Hartung correction for false discovery rate, the prediction interval and heterogeneity values (I^2, tau^2). Contour-enhanced funnel plot showing effect sizes, standard errors, and significance. P-curve analysis, showing the accumulation of p-values over the significant studies (observed p-curve), the no-effect curve and 33% power curve. GOSH (Graphic Display of Heterogeneity) shows distribution for all 2^{k-1} possible study combinations (1 million randomly selected models when $2^{k-1} > 10^6$) in blue and leaving out the most negatively influential study in green.

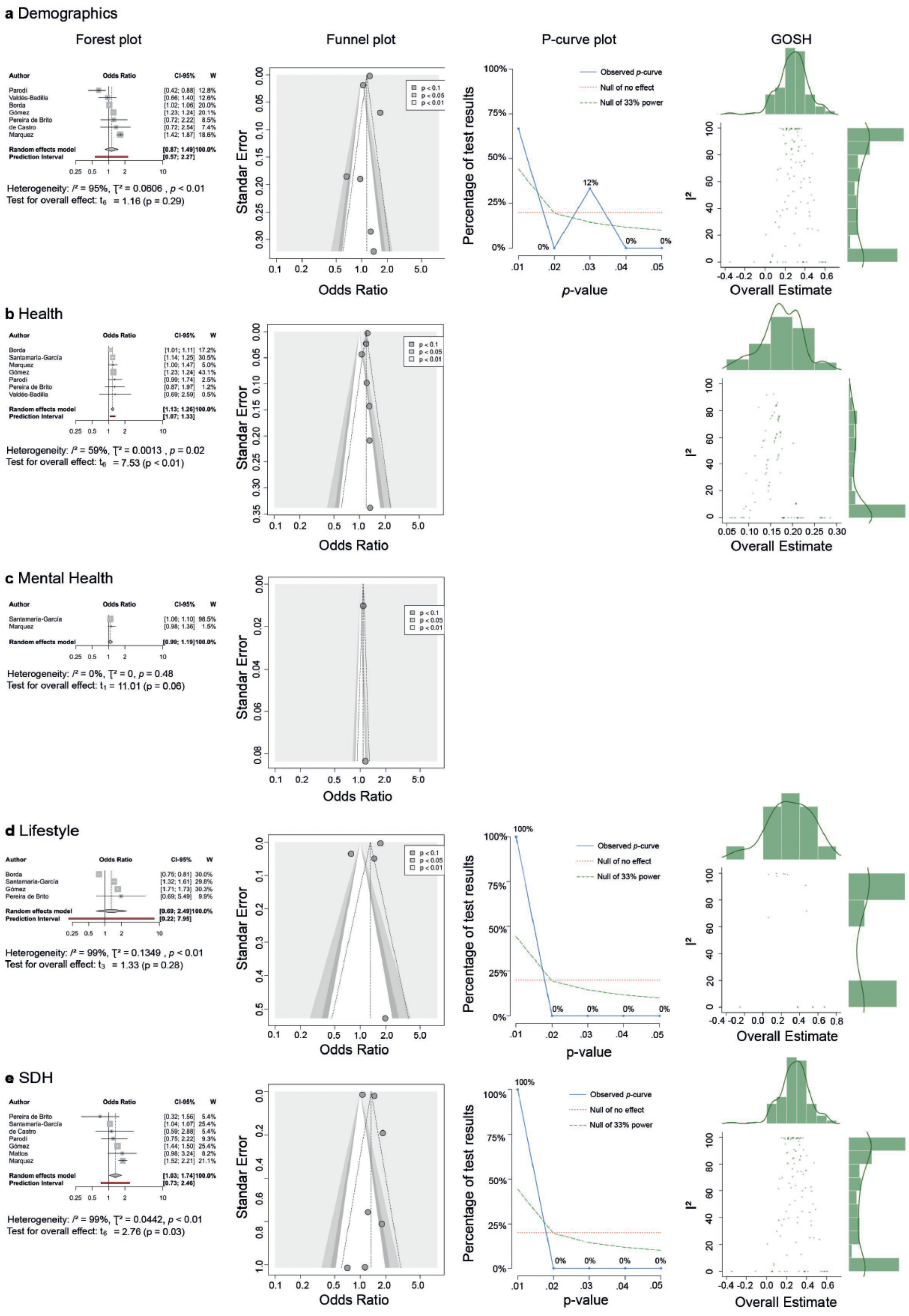

Supplementary Figure 3: Functional ability without outliers. Each panel illustrates the major effects for each factor analyzed: **panel a** focuses on demographics; **panel b** on health; **panel c** on mental health; **panel d** on lifestyle; and **panel e** on Social Determinants of Health (SDH). Blank panels indicate that P-Curves and GOSH cannot be estimated due to insufficient studies. Forest plot shows the studies in random effects model (first author, Odd Ratio, Confidence Interval, and weights). The random effects

model results (Demographics: $k = 7$, $n = 99120$, $OR = 1.137$, *p-value* = 0.2887, $CI = [0.8681; 1.4891]$; Health: $k = 7$, $n = 98992$, $OR = 1.1921$, *p-value* = 0.0003, $CI = [1.1260; 1.2622]$; SDH: $k = 7$, $n = 98793$, $OR = 1.3401$, *p-value* = 0.0328, $CI = [1.0338; 1.7371]$. No analyses were conducted on mental health and lifestyle factors as a consequence of a reduced number of studies.) are reported with Knapp-Hartung correction for false discovery rate, the prediction interval and heterogeneity values (I^2, tau^2). Contour-enhanced funnel plot showing effect sizes, standard errors, and significance. P-curve analysis, showing the accumulation of p-values over the significant studies (observed p-curve), the no-effect curve and 33% power curve. GOSH (Graphic Display of Heterogeneity) shows distribution for all 2^{k-1} possible study combinations (1 million randomly selected models when $2^{k-1} > 10^6$) in blue and leaving out the most negatively influential study in green. Blank panels indicate that P-Curves and GOSH cannot be estimated due to the lack of sufficient studies.
