# Supplementary material for: Healthy aging meta-analyses and scoping review of risk factors across Latin America reveal large heterogeneity and weak predictive models
Source: Nat Aging. 2024 Jun 17;4(8):1153–65. doi: 10.1038/s43587-024-00648-6 (PMC11333291; doi:10.1038/s43587-024-00648-6)
Supplement: Supplementary file 2 — Reporting Summary [file 43587_2024_648_MOESM2_ESM.pdf]

nature portfolio

Corresponding author(s): Agustin Ibanez and Hernando Santamaria Garcia

Last updated by author(s): 2024-03-26

Reporting Summary

Nature Portfolio wishes to improve the reproducibility of the work that we publish. This form provides structure for consistency and transparency in reporting. For further information on Nature Portfolio policies, see our Editorial Policies and the Editorial Policy Checklist.

Statistics

For all statistical analyses, confirm that the following items are present in the figure legend, table legend, main text, or Methods section.

n/a	Confirmed	
☐	☒	The exact sample size (*n*) for each experimental group/condition, given as a discrete number and unit of measurement
☐	☒	A statement on whether measurements were taken from distinct samples or whether the same sample was measured repeatedly
☐	☒	The statistical test(s) used AND whether they are one- or two-sided<br>*Only common tests should be described solely by name; describe more complex techniques in the Methods section.*
☐	☒	A description of all covariates tested
☐	☒	A description of any assumptions or corrections, such as tests of normality and adjustment for multiple comparisons
☐	☒	A full description of the statistical parameters including central tendency (e.g. means) or other basic estimates (e.g. regression coefficient) AND variation (e.g. standard deviation) or associated estimates of uncertainty (e.g. confidence intervals)
☐	☒	For null hypothesis testing, the test statistic (e.g. *F*, *t*, *r*) with confidence intervals, effect sizes, degrees of freedom and *P* value noted<br>*Give P values as exact values whenever suitable.*
☒	☐	For Bayesian analysis, information on the choice of priors and Markov chain Monte Carlo settings
☒	☐	For hierarchical and complex designs, identification of the appropriate level for tests and full reporting of outcomes
☐	☒	Estimates of effect sizes (e.g. Cohen's *d*, Pearson's *r*), indicating how they were calculated

Our web collection on statistics for biologists contains articles on many of the points above.

Software and code

Policy information about availability of computer code

Data collection	Not software was used. Data included in this study is available in https://github.com/AI-BrainLat-team/Latam-Aging-Meta-Analysis/tree/main/Data
Data analysis	All models and statistical analyses were run using Python version 3.9.13. Code is avaiable in GitHub at https://github.com/AI-BrainLat-team/Latam-Aging-Meta-Analysis

For manuscripts utilizing custom algorithms or software that are central to the research but not yet described in published literature, software must be made available to editors and reviewers. We strongly encourage code deposition in a community repository (e.g. GitHub). See the Nature Portfolio guidelines for submitting code & software for further information.

Data

Policy information about availability of data

All manuscripts must include a data availability statement. This statement should provide the following information, where applicable:

- Accession codes, unique identifiers, or web links for publicly available datasets
- A description of any restrictions on data availability
- For clinical datasets or third party data, please ensure that the statement adheres to our policy

Databases used in this study included MEDLINE; EMBASE;Virtual health library, and Web of science

Data availability
Data included in this study is available in GitHub at https://github.com/AI-BrainLat-team/Latam-Aging-Meta-Analysis/tree/main/Data

Research involving human participants, their data, or biological material

Policy information about studies with human participants or human data. See also policy information about sex, gender (identity/presentation), and sexual orientation and race, ethnicity and racism.

Reporting on sex and gender	We conducted a meta-analysis and scoping review. We identified and reported sex diferences, but the reviewed studies did not provide any information on gender identification.
Reporting on race, ethnicity, or and other socially relevant groupings	We conducted a meta-analysis and scoping review. We identified and reported demographic information on ethnicity race factors.
Population characteristics	We conducted a meta-analysis and scoping review on risk factors associated with healthy aging in older age Latin American population.
Recruitment	We described methods of recruitment of studies assessed in our scoping review and meta analysis.
Ethics oversight	Ethic committee from Universidad Javeriana, Bogota,. Colombia approved this study.

Note that full information on the approval of the study protocol must also be provided in the manuscript.

Field-specific reporting

Please select the one below that is the best fit for your research. If you are not sure, read the appropriate sections before making your selection.

☒ Life sciences ☐ Behavioural & social sciences ☐ Ecological, evolutionary & environmental sciences

For a reference copy of the document with all sections, see nature.com/documents/nr-reporting-summary-flat.pdf

Life sciences study design

All studies must disclose on these points even when the disclosure is negative.

Sample size	This study analyzed data from a total of n=146,000 participants. This sample size was taken from all studies meta-analyzed in this study-
Data exclusions	No information was excluded. N/A.
Replication	This is a scoping review and metaanalysis study. We did not implemented new research paradigms but followed international criteria for running this kind of studies.
Randomization	This is a scoping review and metaanalysis study. We did not implemented new research paradigms but followed international criteria for running this kind of studies.
Blinding	Three different researchers assessed the studies included in the meta-analyses and scoping review independently and blinded to the other researchers' reviews. After this review, the reviewers included the studies for meta-analyses and discussed with each other some studies with disparate initial assessments.

Reporting for specific materials, systems and methods

We require information from authors about some types of materials, experimental systems and methods used in many studies. Here, indicate whether each material, system or method listed is relevant to your study. If you are not sure if a list item applies to your research, read the appropriate section before selecting a response.

Materials & experimental systems

n/a	Involved in the study
☒	☐ Antibodies
☒	☐ Eukaryotic cell lines
☒	☐ Palaeontology and archaeology
☒	☐ Animals and other organisms
☒	☐ Clinical data
☒	☐ Dual use research of concern
☒	☐ Plants

Methods

n/a	Involved in the study
☒	☐ ChIP-seq
☒	☐ Flow cytometry
☒	☐ MRI-based neuroimaging

Plants

Seed stocks	N/A
Novel plant genotypes	N/A
Authentication	N/A
